# Virtual memory CD8⁺ T cells in aging: heterogeneity, bystander protection, inflammatory potential, and inhibitory regulation

**DOI:** 10.1186/s12979-026-00582-8

**Published:** 2026-07-10

**Authors:** Archit Kumar, Raymond Yung

**Affiliations:** Department of Internal Medicine, Division of Geriatric and Palliative Medicine, University of Michigan Medical School, Ann Arbor, MI USA

**Keywords:** Aging, CD8^+^ T cells, Memory T cells, Virtual memory T cells, Granzymes, Inflammation

## Abstract

Aging reshapes the CD8⁺ T cell compartment through contraction of the naïve pool and expansion of memory-phenotype populations, including the progressive accumulation of antigen-inexperienced virtual memory (VM) CD8⁺ T cells. VM CD8⁺ T cells arise in the absence of foreign antigen priming and acquire a memory-like phenotype. In aged hosts, VM CD8⁺ T cells constitute a substantial fraction of the antigen-inexperienced memory-phenotype CD8⁺ T cell pool, and their differentiation is shaped by self-reactivity, homeostatic cytokines, and transcriptional programs linked to IL-15 and EOMES. During aging, VM CD8⁺ T cells accumulate numerically, display altered proliferative responses, retain sensitivity to homeostatic and inflammatory cytokines, and can preserve effector function during infection. These features suggest that VM CD8⁺ T cells may partially compensate for the age-associated loss of naïve CD8⁺ T cells by providing rapid effector-like responses. At the same time, aged VM CD8⁺ T cells appear to comprise heterogeneous subsets, including populations with cytotoxic and inflammatory potential. Emerging evidence suggests that inhibitory receptors may mark distinct VM states during aging, although their functional significance and potential regulatory role remain to be established.

## Background

 Aging is associated with a loss of physiological function and dysregulation of the immune system, which increases susceptibility to infections [1–3]. CD8^+^ T cells play an important role in immune surveillance and release IFN-γ and granzymes to mediate killing of the infected cells [4–6]. Aging is accompanied by remodeling of the CD8⁺ T cell compartment, characterized by contraction of the naïve pool and accumulation of memory-phenotype T cells, resulting in a reduced T cell repertoire associated with impaired response to infections [7–12]. Moreover, CD8^+^ T cells have been implicated as one of the factors contributing to the chronic low-grade inflammation termed inflammaging [3, 13–15].

Conventional memory CD8^+^ T cells, which arise following antigen exposure, are broadly divided into effector memory, central memory and tissue resident subsets, based on their function, localization, longevity, and phenotypic markers [16–24]. However, in antigen-unprimed hosts, a significant pool of CD8⁺ T cells bearing a memory phenotype exists alongside the naïve compartment [16, 25–27]. These antigen-inexperienced memory CD8^+^ T cells are categorized into innate memory (IM) and virtual memory (VM) cells, which differ by their developmental origin, localization and cytokine dependence [16, 25–27]. IM CD8⁺ T cells arise predominantly in the thymus and are shaped by IL-4 produced by PLZF-expressing thymocytes [26, 28–34]. By contrast, VM CD8⁺ T cells arise mainly in the periphery from naïve CD8⁺ T cells with stronger tonic self-peptide–MHC recognition, often reflected by high CD5 expression, and are maintained through IL-15/CD122-dependent homeostatic signaling [26, 35–38]. Although both IM and VM CD8⁺ T cells can express CD44, CD122, and EOMES and generally lack CD49d, they are best distinguished from each other by thymic IL-4-dependent programming versus peripheral IL-15-dependent homeostatic differentiation [25–27, 37–39]. Although both populations depend on IL-15 in the periphery, their anatomical distribution differs. IM cells are predominantly found in secondary lymphoid organs such as the spleen following thymic egress, whereas VM cells recirculate through blood and secondary lymphoid organs and additionally accumulate in non-lymphoid tissues including the liver, lung, and adipose tissue [26, 27, 36, 40–43]. In the periphery, VM cells constitute 5–20% of CD8^+^ T cells and express high levels of CD44, CD62L, CD122/IL-15Rβ, EOMES, and rely on cytokines for their survival, maintenance, and proliferation [16, 25–27]. While VM CD8⁺ T cells in young mice exhibit effector functions and mediate bystander protection, they exhibit proliferation defects during aging [36, 39, 44, 45]. Moreover, emerging data suggest that VM CD8^+^ T cells constitute heterogeneous subsets, express inhibitory receptors, and can mediate inflammatory diseases, with similar characteristics identified in aged hosts through single-cell-based analyses [42, 46–48].

**Fig. 1:**
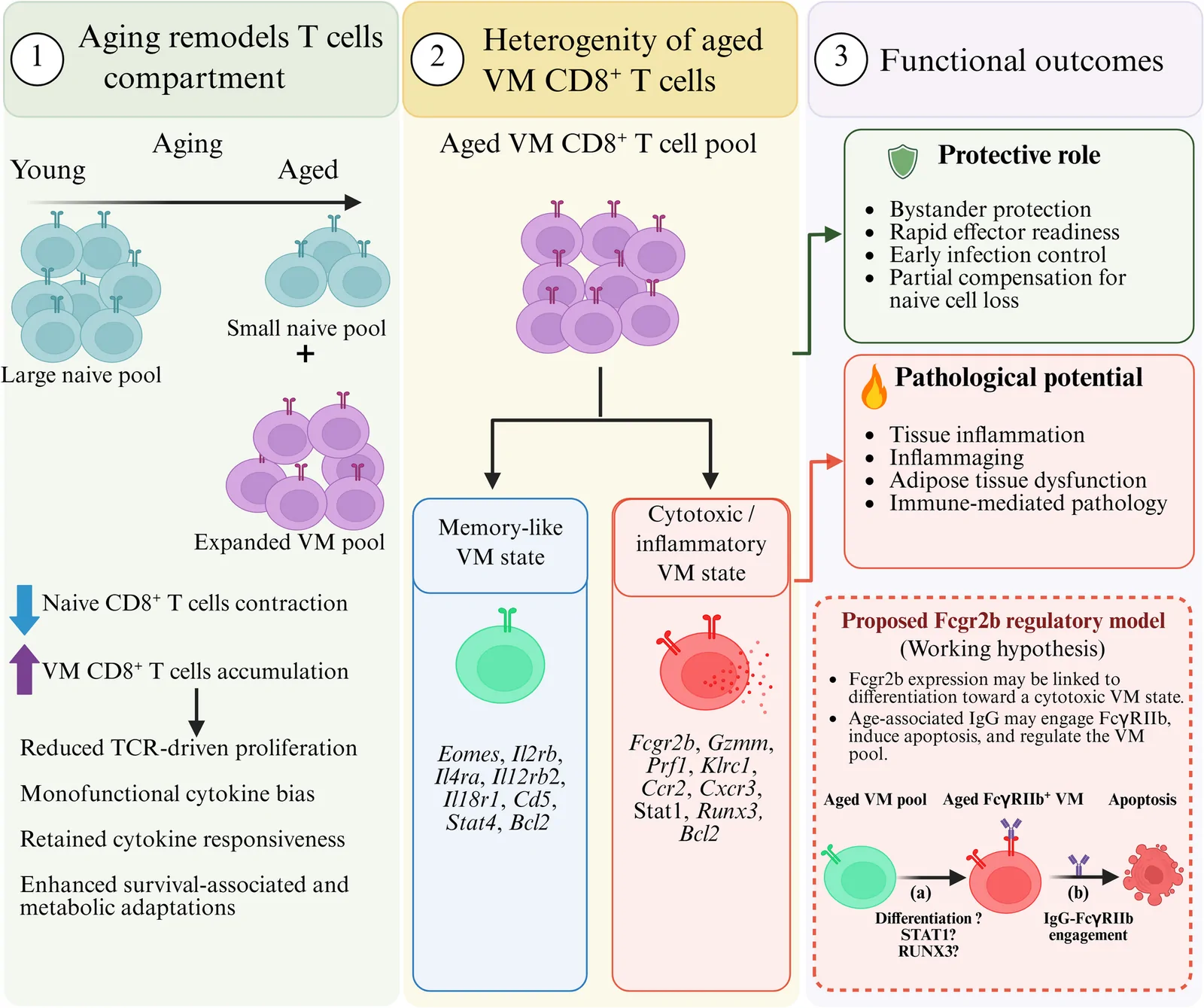
Aging-driven remodeling and regulatory heterogeneity of VM CD8^+^T cells. VM CD8^+^ T cells arise from antigen-inexperienced naïve CD8 ^+^ T cells under the influence of tonic self-pMHC/TCR signaling, self-reactivity, IL-15, context-dependent IL-4, and EOMES-dependent programming. During aging, contraction of the naïve CD8 + T cell pool is accompanied by accumulation of VM CD8^+^ T cells, which retain cytokine responsiveness but exhibit impaired TCR-driven proliferation together with survival and metabolic fitness. Emerging evidence suggests that the aged VM compartment is heterogeneous and includes at least two broad states: a memory-like/homeostatic VM state characterized by *Eomes*, *Il2rb* (CD122), *Bcl2* survival-associated features, and rapid responder potential (*Il12rb2*, *Il18r1*), and a cytotoxic/inflammatory VM state enriched for *Fcgr2b*, *Gzmm*, *Ccr2*,* Cxcr3*, *Stat1,* and *Runx3*. A proposed working model is that FcγRIIb may contribute to differentiation or stabilization of the cytotoxic VM state, whereas age-associated IgG may engage FcγRIIb and promote apoptosis or restrain persistence of the Fcgr2b+ subset. Functionally, aged VM CD8^+^ T cells may provide beneficial early effector-like responses and partial compensation for naïve CD8^+^ T cell loss, but may also contribute to chronic tissue inflammation, adipose dysfunction, and age-associated inflammatory disease. Further mechanistic studies are required to define the role of FcγRIIb in VM cell fate and function. The model described in Sect. 2 is based on single-cell RNA-seq, RNA velocity, and Monocle pseudotime trajectory analyses of aged adipose tissue dataset reported in Kumar et al. [42], which is openly available in the Gene Expression Omnibus (GEO) under accession number GSE300036

In this review, we discuss the development, function, heterogeneity, bystander protection, and regulatory potential of VM CD8⁺ T cells, with particular emphasis on their remodeling during aging and their emerging role in age-associated inflammation (Table 1). We also propose a working model in which the B-cell-associated inhibitory receptor Fcgr2b may contribute to the regulation of the aged VM compartment (Fig. 1).

**Table 1:** Phenotypic and functional remodeling of VM CD8^+^ T cells during aging

Biological features	Young VM CD8^+^ T cells (mouse)	Aged VM CD8^+^ T cells (mouse)	Human VM-like CD8^+^ T cells	References
Differentiation and transcriptional state	EOMES⁺ IL-15-dependent homeostatic programming	EOMES is maintained, whereas aged VM-like states can express Gzmm, Ccr2, Cxcr3, Runx3, Stat1 and Fcgr2b in cytotoxic subsets	EOMES⁺ and Nur77⁺ features support partial conservation of IL-15 responsiveness and basal TCR signaling, cytotoxic VM-like transcriptomic states remain less well defined in human aging	[36, 42, 45, 146]
Homeostatic cytokine responsiveness	IL-15-dependent maintenance and proliferation	Homeostatic cytokine responsiveness, particularly to IL-15, is preserved despite impaired TCR-driven proliferation	CD122⁺ VM-like cells are highly responsive to IL-15 comparable to murine VM cells: co-expressing Tbet and IFN-γ production	[36, 105, 107, 146]
TCR-driven proliferation	Enhanced antigen responsiveness following TCR stimulation	Impaired after TCR engagement, with reduced clonal expansion, increased apoptosis and acquisition of senescence-associated signaling	Reduced TCR-driven proliferation has been reported in aged human VM-like CD8⁺ cells, resembling the proliferative defects described in aged murine VM cells	[39, 45, 105, 107, 109]
Cytokine polyfunctionality (IFN-γ, TNF-α, Granzymes)	Polyfunctional IFN-γ and TNF-α co-production, rapid GzmB upregulation upon IL-12/IL-18 Or Rapid IFN-γ production after IL-12/IL-18 stimulation	Shift toward monofunctional cytokine production, GzmB and GzmM retained in cytotoxic subsets	Rapid IFN-γ production upon IL-12 + IL-18 stimulation, full polyfunctionality not characterized	[43, 107, 146]
Bystander / innate-like effector capacity	NKG2D-mediated bystander cytotoxicity, IL-12/IL-18-driven IFN-γ, protection against *Listeria monocytogenes*	Retained innate-like effector capacity during infection, major contributor to the antiviral response in aged influenza-naïve mice	Rapid IFN-γ upon IL-12 + IL-18, innate-like cytotoxicity documented in alopecia areata, CD62L⁺CD45RO⁺CD25⁺ protection against influenza	[36, 43, 44, 48, 150, 151]
Senescence	Low γ-H2AX, low p-p38 / p-ERK1/2, minimal MAPK pathway activation	Elevated γ-H2AX, p-p38 MAPK, p-ERK1/2, senescence-like state, attenuated by prior LCMV infection	Not fully defined, although impaired proliferation in aged donors suggests partial convergence with senescence-like biology.	[107, 109, 111]
Metabolic and survival adaptations	Supported by IL-15-dependent survival programs and baseline mitochondrial and glycolytic fitness.	Displays high spare respiratory capacity, preserved glycolytic potential and enhanced anti-apoptotic programming, including Bcl2-family-associated survival features	Aged human VM-like cells can exhibit high spare respiratory capacity; however, metabolic fitness does not necessarily predict antigen-driven function.	[42, 107–109]
Subset heterogeneity	Includes functionally distinct subsets, including CXCR3 expressing CCR2⁺ effector-biased cells and CCR2⁻ memory-precursor-like cells that can contribute to tissue responses	Additional cytotoxic VM-like states have been described in aged adipose tissue, including Gzmm⁺ Fcgr2b⁺ Ccr2⁺ Cxcr3⁺ Runx3⁺ Stat1⁺ cells.	Whether comparable VM-like CD8⁺ T cell heterogeneity exists in humans, particularly in aged individuals, remains unclear and will require further investigation	[42, 46, 47]
Inhibitory and/or regulatory receptors	CD22 can restrain IL-4-driven VM expansion and activation during helminth infection	Elevated Fcgr2b on Cytotoxic Tvm subset. Although Fcgr2b expression is elevated in the cytotoxic Tvm subset, its role in regulating VM CD8⁺ T cell abundance, differentiation, or effector function remains to be defined.	KIR^+^ and NKG2A^+^ mark proposed human VM-like populations, functional significance in aging not established and their relationship to murine Fcgr2b⁺ cytotoxic VM states remains unknown.	[42, 44, 46, 145, 146]
Effects of infection history	Helminth-induced IL-4 can expand VM CD8⁺ cells, IL-15-dependent expansion, and enhance antiviral readiness in young mice	Aged VM cells fail to expand following helminth infection, LCMV infection depletes the VM pool but enriches for TCR-sensitive survivors with attenuated senescence	CMV and other lifelong infections likely confound VM-like phenotypes by CD45RA reexpression, CD57⁺, CD27^−^or antigen-experienced CD8⁺ populations, VM-like assignment therefore requires careful exclusion of antigen-specific expansions	[96], 110– [112, 115], 117– [119, 121]
Role in inflammation	Primarily linked to early bystander protection rather than chronic sterile inflammation under steady-state conditions	Cytotoxic VM-like subsets may contribute to granzyme-associated tissue inflammation and age-associated adipose immune remodeling	Human VM-like cytotoxic CD8⁺ cells have been implicated in alopecia areata, their broader contribution to inflammaging remains unresolved	[14, 42, 48]

## Defining VM CD8^+^ T cells

Conventional antigen-driven CD8^+^ memory cells are mainly described as effector memory (EM) and central memory (CM) T cells [16, 49, 50]. Both populations express the activation-associated receptor CD44, but EM CD8^+^ T cells are CD62L^−^ CCR7^−^, whereas CM CD8^+^ T cells express homing receptors CD62L and CCR7, which direct their localization in lymphoid tissues [16, 23, 51]. Functionally, EM CD8^+^ T cells are more immediately poised for effector function, whereas CM CD8^+^ T cells are long-lived and exhibit greater self-renewal and proliferative capacity. Both EM and CM CD8^+^ T cells depend on common γc cytokines, IL-7 and IL-15, for homeostatic maintenance; however, CM CD8⁺ T cells rely more heavily on IL-7, owing to their higher IL-7Rα (CD127) expression and CCR7/CD62L-dependent localization to secondary lymphoid organs, which support memory T cell survival and homeostatic proliferation via IL-7 and IL-15 [16, 52–54].VM CD8^+^ T cells share several surface and transcriptional features with conventional antigen-experienced memory CD8⁺ T cells, including expression of CD44, CD122/ IL-15Rβ, and EOMES [25–27]. These shared features reflect a common dependence on IL-15 signaling and common memory-like transcriptional programming under homeostatic proliferation. However, unlike true antigen-experienced memory cells, VM CD8^+^ T cells are defined by the lack of CD49d (encoded by *Itga4*), a marker associated with prior antigen encounter [39]. Accordingly, VM CD8^+^ T cells are most precisely described by the surface phenotype CD44^+^ CD62L^+^ CD122^+^ CD49d^−^ [25–27, 39, 55]. VM CD8^+^ T cells can also display rapid functional responsiveness to inflammatory cytokines, including IL-12 and IL-18, with induction of IFN-γ and expression of cytokine-responsive genes such as Il18r1, Il12rb1, and Il12rb2 [27, 39]. VM CD8^+^ T cells share several features with thymus-derived IM CD8^+^ T cells in circulation, however, IM cells are generally less associated with effector-linked programs, and can be distinguished from VM cells by lower surface NKG2D expression, consistent with the greater cytotoxic bias often attributed to VM cells [25, 26, 56, 57]. Notably, while thymic IM cells lack T-bet expression, peripheral IM cells upregulate T-bet following IL-15 exposure, rendering T-bet unreliable as a distinguishing marker between circulating IM and VM cells [26]. Although phenotypic definitions remain useful, VM CD8^+^ T cells should be interpreted with caution in the setting of infection, because antigen-specific CD8⁺ T cells can lose CD49d expression over time, complicating discrimination from bona fide VM CD8^+^ T cells [58].

### Developmental origins of antigen-inexperienced memory-like CD8^+^ T cells

#### Conventional CD8^+^ T cell development

CD8^+^ T cells develop in the thymus, where immature thymocytes undergo sequential differentiation and selection steps to generate a functional and self-tolerant T cell receptor repertoire [59, 60]. During this process, double-negative thymocytes undergo β-selection, followed by positive and negative selection based on strength of recognition of self-peptide-MHC complexes [59, 60]. These signals not only ensure self-tolerance but also guide lineage commitment toward either the CD4⁺ or CD8⁺ T cell lineage [59, 61]. As CD8 single positive (SP) thymocytes mature, they upregulate CCR7 and CD62L, egress from the thymus, and enter the periphery as naïve CD8⁺ T cells [62–65]. Upon cognate antigen encounter, these naïve CD8^+^ T cells become activated, differentiate into effector cells, and eventually give rise to conventional memory CD8^+^ T cell phenotypes known as EM and CM T cells [16, 18].

Conventional memory arises after antigen exposure and is typically maintained in secondary lymphoid organs and peripheral tissues, and is associated with expression of CD44, CD62L, and the transcription factor EOMES [16, 18, 66, 67]. However, it is now clear that not all CD8^+^ T cells with a memory phenotype are generated through classical antigen-driven priming [26]. In addition to conventional memory cells, there are antigen-inexperienced memory-like CD8^+^ T cell populations, including IM CD8^+^ T cells that develop in the thymus and VM CD8⁺ T cells that arise in the periphery [25, 26, 68, 69].

### Thymic antigen-inexperienced IM CD8^+^ T cells

Innate memory CD8⁺ T cells were first described in the thymus of mice lacking the TEC kinase IL-2–inducible T cell kinase (ITK), where they were identified by the expression of markers such as CD44 and CD122 [31–34]. Later studies showed that the accumulation of these cells is not simply due to a CD8⁺ T cell–intrinsic program but is strongly influenced by IL-4 produced largely by PLZF-expressing cells, including natural killer T (NKT) cells, and CD4⁺ T cells [26, 28, 30, 56, 57]. Their accumulation is also strongly influenced by background strain: BALB/c mice generate substantially more IM CD8^+^ T cells than C57BL/6 mice [26, 29, 34, 70]. However, it remains unclear whether the conversion of single positive CD8⁺ T cell into IM in the thymus is stochastic or depends on specific stimuli.

### Peripheral VM CD8^+^ T cells

In contrast to thymic IM CD8^+^ T cells, VM CD8⁺ T cells develop mainly outside the thymus and are maintained in the periphery [26, 37, 38, 68]. They are highly responsive to IL-4 and IL-15 for their development and proliferation [37, 71]. Unlike thymic IM CD8^+^ T cells, which rely heavily on IL-4, VM CD8⁺ T cells can arise in the absence of IL-4 in C57BL/6 mice whereas in BALB/c mice VM development is IL-4-dependent [26, 37, 38]. VM CD8⁺ T cells are strongly dependent on IL-15 and EOMES for their expansion and maintenance and are therefore absent in IL-15- and CD122-deficient mice [26, 37, 38]. Under steady-state, VM CD8⁺ T cells rely on basic leucine zipper transcription factor ATF-like 3 (BATF3)-dependent CD8α^+^ dendritic cells (DCs) as the major source of IL-15 [37, 72]. Although IL-15 plays a crucial role in the proliferation and maintenance of VM CD8^+^ T cells, IL-7 may contribute by sustaining the naïve CD8^+^ T cell pool from which VM CD8^+^ T cells arise. Homeostatic proliferation of naïve CD8^+^ T cells is driven by IL-7 together with tonic TCR-self-pMHC signals [73]. However, naïve CD8^+^ T cells can further be supported by elevated levels of γc cytokines including IL-7, memory CD8^+^ T cells rely mainly on IL-7 or IL-15 [73–77]. Together, VM CD8^+^ T cell development reflects the intersection of naïve CD8 + T cells homeostasis, self-pMHC-driven fitness and IL-15-depedendent peripheral expansion, rather than a single cytokine-driven pathway [36–38].

### Transcriptional regulation

Transcriptional regulation mediated by T-box transcription factors plays a crucial role in CD8^+^ T cell memory development. The balance between T-bet and EOMES determines effector and memory fate: T-bet favors differentiation of terminal effectors [78, 79], while EOMES promotes the development of long-lived central memory CD8⁺ T cells and their survival following homeostatic proliferation [40, 66, 80, 81]. IL-4-mediated expression of EOMES is essential for the development of IM CD8⁺ T cells from CD8 SP thymocytes [29, 82, 83], a process involving EOMES interaction with RUNX3-bound enhancers in a BRG1-dependent manner [84]. In VM CD8⁺ T cells, EOMES is required for effective IL-15-mediated homeostatic proliferation and upregulates the expression of CD122/IL-15Rβ [37]. IL-4 and type I interferons further regulate EOMES-mediated expansion of VM CD8⁺ T cells [71, 72], while Aiolos (Ikzf3)-mediated repression of EOMES constrains VM CD8⁺ T cell generation [85].

### Tonic TCR signaling and self-reactivity

T cell receptor (TCR) signaling is a key determinant of cell fate for VM CD8^+^ T cell differentiation [35], while thymic IM CD8 + T cells development depends primarily on IL-4 produced by PLZF-expressing iNKT and/or CD4^+^ cells [26]. Naïve CD8⁺ T cells with stronger tonic TCR signaling in response to self-peptide–MHC are more likely to differentiate into VM CD8^+^ T cells [35]. Consistent with this, naïve CD8^+^ T cells with high CD5 expression are more likely to convert into VM CD8^+^ T cells than those with low CD5 expression [35, 36]. CD5 is used as a marker of self-reactivity, and its expression reflects the strength of signaling upon engagement of TCR with self-peptide-MHC. VM CD8⁺ T cells are enriched among naïve CD8⁺ T cell clones with stronger tonic recognition of self-peptide–MHC, suggesting that basal TCR signal strength may influence their entry into a VM-like state [35, 36, 86]. In support of this idea, CD8–Lck coupling has been shown to affect tonic TCR signaling and VM CD8⁺ T cell formation, although this mechanism should be interpreted as contributing to VM-cell generation or enrichment rather than peripheral expansion [35, 36, 86].

## Functional programming of VM CD8^+^ T cells

### Innate-like effector capacity and bystander protection

Homeostatic proliferation (HP) can generate memory-phenotype CD8⁺ T cells in the unprimed host [87–91], and these HP memory cells display greater proliferative, cytotoxic, cytokine-producing capacity than naïve T cells, including protection following infection [89–93]. Together, these observations suggest that antigen-inexperienced memory CD8⁺ T cells are functionally poised for rapid innate-like effector responses. Consistent with this, Haluszczak et al. identified antigen-specific CD44hi CD8⁺ T cells in unprimed mice that exhibited enhanced proliferative responses to antigen relative to naïve precursors and rapidly produced IFN-γ following stimulation with IL-12 and IL-18 [39]. Lee et al. subsequently demonstrated that naturally occurring polyclonal VM CD8⁺ T cells constitute a functionally distinct antigen-inexperienced population [45]. Although these cells respond more rapidly than naïve precursors and can mediate protection against *Listeria monocytogenes* (LM), they are not functionally equivalent to bona fide antigen-experienced memory CD8⁺ T cells, as they exhibit limited TCR-driven IFN-γ production [45]. Later studies further strengthened this framework by showing innate-like effector function of VM CD8^+^ T cells, which are poised for bystander protection and express IL-12R, IL-18R, IFN-γ, NKG2D, and granzyme B, consistent with inflammatory responsiveness [36, 44]. Bystander protection mediated by VM CD8^+^ T cells against LM is strongly IL-15 dependent, as loss of IL-15 markedly reduced expression of IFN-γ, NKG2D, and granzyme B in VM CD8^+^ T cells [36]. The same study also showed that their distinct trafficking properties help determine where this protection can occur by constraining tissue access and localization [36]. Further dissecting the heterogeneity within VM CD8^+^ T cells, Hou et al. identified a CCR2⁺ subset within VM CD8^+^ T cells that is preferentially poised for effector function, expresses cytotoxicity-associated genes including Gzmm and Ctla2a, rapidly infiltrates the lung through CXCR3-mediated recruitment during primary influenza infection, and promotes early viral control [47].

### Cytokine- and infection-driven plasticity

Beyond steady-state homeostatic maintenance, cytokine sensitivity and inflammatory stimuli further shape the effector potential of antigen-inexperienced memory-phenotype CD8⁺ T cells. Renkema et al. showed that IL-4 responsiveness influences both the size and functional composition of the peripheral memory-phenotype CD8⁺ T cell pool and contributes to a robust response following LCMV infection [94]. In a related but distinct setting, Baez et al. showed that Th1-biased infection, as well as IL-12 plus IL-18 administration induces thymic IL-4 and IL-15 and promotes the emergence of single-positive CD8 thymocytes with innate-like features; adoptive transfer of these cells improved control of *Trypanosoma cruzi* infection, indicating that inflammatory conditions can imprint innate effector properties during thymic CD8⁺ T cell development [95].

Helminth infection provides one of the clearest examples of cytokine-driven VM plasticity. Helminth-induced IL-4 response in BALB/c mice has been shown to expand the VM CD8^+^ T cells compartment and enhance protection against murid gammaherpesvirus-4 [96]. This expansion was associated with increased expression of cytotoxic genes (*Gzma*, *Gzmm*), chemokine receptor genes (*Ccr2* and *Ccr5*), and type I interferon-related gene *Mx1*. Following viral challenge, helminth-infected mice also exhibited enhanced expansion of virus-specific CD8⁺ T cells and increased frequencies of TNF-α- and IFN-γ-producing CD8⁺ T cells [96]. Extending this framework, Yang et al. used single-cell RNA sequencing to identify a helminth-induced, IL-4-responsive CD22⁺ VM CD8^+^ T cell subset. Notably, this population was enriched for effector-associated genes, including Ifng, Gzma, Gzmm, Ccr2, Ccr5, and Cd39 [46].

Mechanistically, antigen-independent bystander activation of VM CD8⁺ T cells is likely to draw on signaling pathways in which IL-12 and IL-18 promote IFN-γ transcription through STAT4- and AP-1-associated programs, whereas IL-15 supports survival, expansion, and granzyme B expression [36, 97–99]. Constitutive expression of IL-12R, IL-18R, and CD122 on VM CD8^+^ T cells along with increased chromatin accessibility at effector genes in these cells [100] poise them for rapid responses during the early phase of infection, particularly when the frequency of naïve precursors is low. Together, these studies indicate that the effector potential of VM CD8^+^ T cells is not fixed but is dynamically tuned by cytokine exposure, inflammation and developmental timing.

### VM CD8^+^ T cells in aging

#### Age-associated accumulation of VM CD8^+^ T cells

VM CD8^+^ T cells emerge during the neonatal period of physiological lymphopenia and persist throughout adulthood [38]. Timestamping analyses have demonstrated that neonatal CD8^+^ T cells are more likely than adult CD8^+^ T cells to undergo homeostatic proliferation and adopt a VM phenotype [100]. During aging, the naïve CD8⁺ T cell compartment undergoes nonrandom attrition, leaving behind an antigen-inexperienced pool enriched for precursors with higher TCR:pMHC avidity [101]. In unprimed aged hosts, these naïve CD8^+^ T cells undergo enhanced homeostatic turnover and acquire a memory-like phenotype, consistent with preferential preservation of clones with superior self-pMHC-driven homeostatic fitness [101]. Following thymic involution, maintenance and proliferation of the antigen-inexperienced CD8^+^ T cell pool become increasingly dependent on peripheral homeostatic signals, including IL-7-dependent survival signals. During aging, increased circulating levels of IL-7 has been reported [102]. While IL-7 may preserve antigen-inexperienced CD8⁺ T cells with sufficient homeostatic fitness, whereas IL-15 could promote proliferation and acquisition of memory- or VM-phenotype during aging [36, 38]. Although age-related accumulation of antigen-inexperienced CD44hi CD8⁺ cells had been reported previously [103], key evidence came from Chiu et al. (2013), who showed that the CM CD8^+^ T cells population that accumulated with age is composed mainly of antigen-inexperienced VM CD8^+^ T cells. Importantly, this age-associated accumulation occurs independently of CD4^+^ T cells, CCR5, and CXCR3 [55] and is accompanied by heightened responsiveness to homeostatic cytokines, driven by upregulated CD122 expression, as well as to type I interferons [72, 104, 105]. Moreover, VM CD8^+^ T cells have also been identified in extralymphoid tissues such as lungs, liver, and aged adipose tissue, suggesting that local tissue environments may redirect them toward pathogen-responsive or metabolically inflammatory states [36, 42, 43]. The tissue distribution of VM CD8⁺ T cells reflect a combination of local survival signals and trafficking-associated programs. In IL-15-rich tissues such as the liver, and potentially in adipose tissue, IL-15 may support the maintenance or accumulation of VM CD8⁺ T cells [26, 36, 106]. During inflammation, VM CD8⁺ T cell entry into peripheral tissues may be supported by chemokine receptor expression and type I interferon-associated inflammatory cues. For example, in young mice, CCR2⁺ VM CD8⁺ T cells rapidly infiltrate the lung during influenza infection and express CXCR3-associated trafficking programs; however, additional studies are needed to determine how these pathways regulate VM CD8⁺ T cell accumulation in aged hosts after infection [47]. High expression of CD44 may further support tissue retention, while LFA-1 (CD11a/CD18) supports adhesion to vascular endothelium and tissue entry [21, 26, 36]. Together, these trafficking-associated properties may direct VM CD8⁺ T cells toward specific compartments such as the liver, lung, and adipose tissue, shaping where they contribute to early immune defense or, in aged hosts, to tissue inflammation.

#### Functional remodeling of aged VM CD8^+^ T cells

Age-associated accumulation of VM CD8^+^ T cells is accompanied by selective functional remodeling rather than uniform loss of responsiveness. As the naïve CD8^+^ T cell pool contracts with age, clones with self-MHC-driven homeostatic fitness are preferentially retained and can remain functionally competent during infection. Rudd et al. demonstrated that high-avidity CD44hi Vβ10⁺ gB-8p-precursors remained enriched during aging and preserved efficient recruitment together with effector differentiation, including KLRG1 upregulation, CD62L downregulation, and maintenance of polyfunctional IFN-γ, TNF-α, and granzyme B production [101]. By contrast, Lanzer et al. directly examined bona fide VM CD8^+^ T cells and showed that, in aged influenza-naïve mice, VM CD8^+^ T cells make a major contribution to the antiviral response, differentiate into granzyme-producing effectors, and support viral clearance [43]. Although aged VM CD8^+^ T cells retain effector responsiveness following TCR-driven stimulation [105, 107], this response in aged VM CD8^+^ T cells becomes increasingly dominated by monofunctional rather than polyfunctional cytokine-producing cells [107]. In parallel, aged VM CD8^+^ T cells exhibit impaired TCR-driven proliferation, linked to increased apoptosis and acquisition of a senescence-like state marked by increased γ-H2AX and phosphorylation of p38, ERK1/2 and MAPK [105, 107]. Notably, this proliferation impairment does not necessarily indicate a complete loss of cell division programs, as aged VM CD8^+^ T cells retain higher frequency of asymmetric cell division than aged naïve CD8^+^ T cells [108]. Although aged VM CD8 + T cells proliferate poorly after TCR engagement, they retain the capacity to proliferate in response to homeostatic cytokines and maintain high expression of CD122/IL-15Rβ [105, 107].

#### Metabolic and survival adaptations of aged VM CD8^+^ T cells

The persistence of aged VM CD8^+^ T cells is supported by intrinsic survival and metabolic programs. Increased expression of anti-apoptotic genes *Bcl2*, *Bcl2l2*, and *Bcl2l1* in aged VM CD8^+^ T cells is associated with their accumulation with age [107]. Metabolically, aged VM CD8^+^ T cells display high spare respiratory capacity, preserved glycolytic capacity and heightened IL-15 responsiveness, consistent with enhanced survival capacity [108, 109]. Yet these adaptations do not rescue their antigen-driven in vitro cytotoxicity and TCR-driven proliferative response [109]. Consistent with this, Borsa et al. showed that aged VM CD8^+^ T cells preserve asymmetric cell division, and that their daughter cells exhibit greater memory potential, improved expansion, and enhanced cytokine production following viral challenge [108].

#### Infection history reshapes aged VM CD8^+^ T cells

As discussed in the previous section ‘Cytokine- and infection-driven plasticity’, helminth infection expands the VM CD8^+^ subset in a cytokine-dependent manner. However, Hussain et al. showed that VM CD8^+^ T cells from aged mice exhibit both intrinsic and extrinsic defects and fail to expand following helminth infection [110]. Extending this line of work, the same group examined how different infections reshape the VM CD8^+^ T cell pool, they further showed that lymphocytic choriomeningitis virus (LCMV) causes a significant loss of VM CD8^+^ T cells, leaving behind a residual VM CD8^+^ T cell pool with higher TCR sensitivity. Notably, this heightened TCR sensitivity persisted into old age and was accompanied by attenuation of senescent VM CD8^+^ T cells [111]. The finding that viral infection can attenuate senescence in aged CD8^+^ T cells further questions the relationship between lifelong infections such as cytomegalovirus (CMV) acquired during aging and VM CD8^+^ T cells. In humans, however, establishing the relationship between persistent viral infection and VM-like CD8⁺ T cells requires caution. Evidence suggests an association between CMV infection and expansion of IL-15-responsive CD57⁺ VM-like CD8⁺ T cells [112–114]. Persistent viral infections, especially CMV, can generate large antigen-specific clonal expansions within the CD8⁺ T cell compartment in elderly individuals [115]. CMV pp65-specific CD8⁺ T cells frequently acquire a terminal or terminally differentiated effector memory (TEMRA)-like phenotype characterized by CD45RA re-expression, CD57 expression, and loss of CD27 and CD28, markers that overlap substantially with proposed human VM-like CD8⁺ T cell phenotypes [112, 113, 115–119]. Moreover, CMV lysate has been shown to induce IL-15 production from leukocytes and stimulation of IL-15 induces expression of CD45RA in CD45RA^−^ CD45RO^+^ CMV-specific CD8^+^ T cells [120]. Therefore, associations between CMV seropositivity and expansion of CD57⁺ and/or CD45RA⁺ cells within human VM-like CD8⁺ T cell populations must be interpreted with caution, because these cells could represent cytokine-expanded antigen-inexperienced cells, antigen-experienced CMV-specific clones, or a mixture of both. Distinguishing these possibilities will require antigen-specific analyses, such as CMV peptide-MHC multimer staining, together with TCR repertoire assessment and CD57 expression as a marker of replicative senescence [121, 122]. Together, these findings suggest that aging does not produce a uniform VM CD8^+^ T cell compartment, rather, it leaves behind a larger but functionally heterogeneous pool whose survival, proliferative fitness, and effector quality can be reshaped by infection history.

### Heterogeneity, inflammation and regulatory potential of aged VM CD8^+^ T cells

#### Heterogeneity and inflammatory potential of VM CD8^+^ T cells

**Fig. 2:**
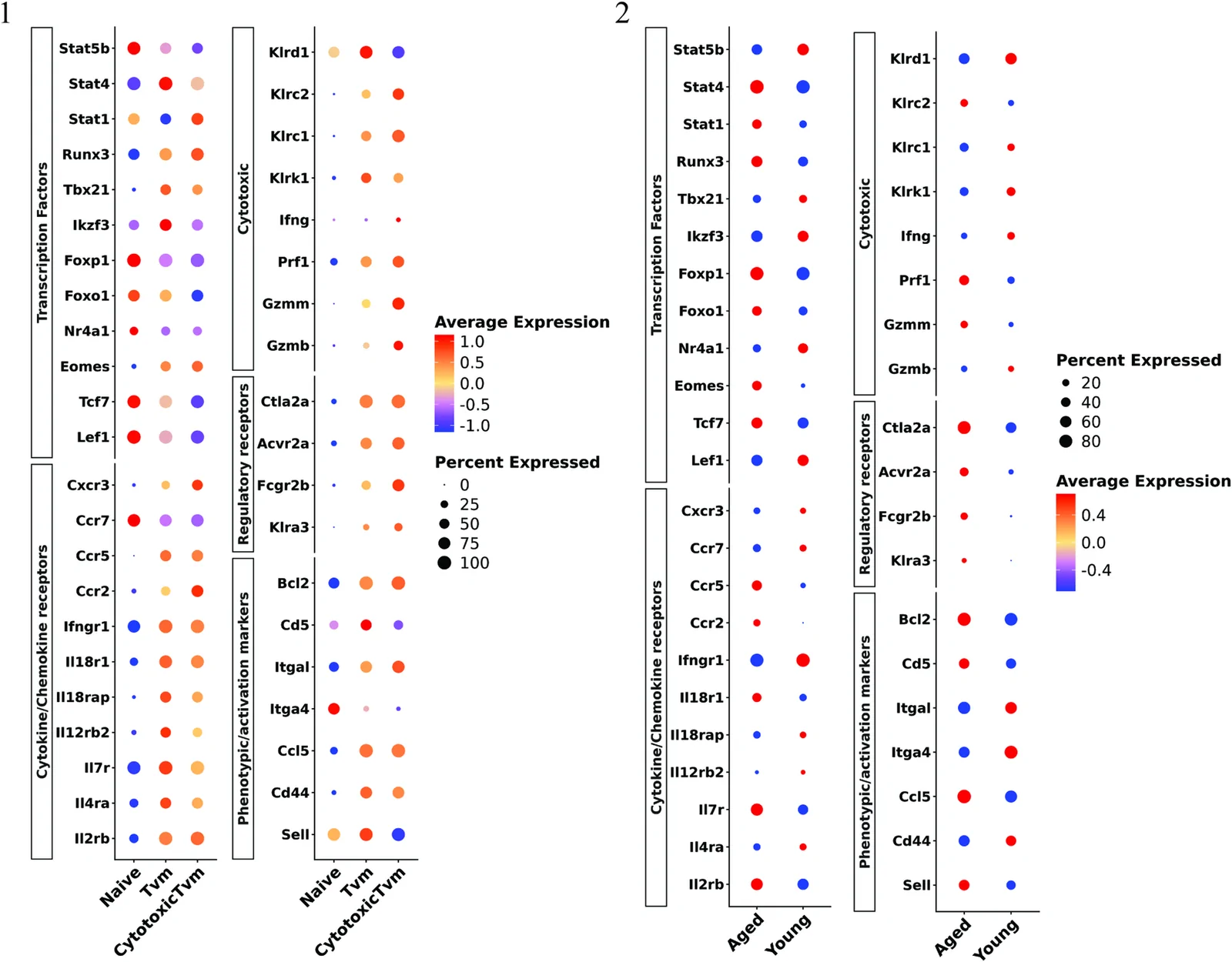
Heterogeneity within aged VM CD8^+^ T cells. (1) Dot plot showing scaled expression of transcription factors, cytokine and chemokine receptors, cytotoxicity-associated genes, inhibitory/regulatory receptors, and phenotypic/activation markers in naïve, Tvm, and cytotoxic Tvm CD8^+^ T cell subsets from aged adipose tissue. (2) Dot plot showing expression of the same marker set in CD8α-expressing cells from young and aged adipose tissue. Dot size indicates the percentage of cells expressing each marker, and color indicates scaled average expression. This dot plot was generated for the present review using the previously published single-cell RNA-sequencing dataset reported in Kumar et al. [42], which is openly available in the Gene Expression Omnibus (GEO) under accession number GSE300036

The VM CD8^+^ T cell compartment is composed of heterogeneous pools that display properties of cytotoxic CD8^+^ T cells. In young mice, VM CD8^+^ T cells differentiate into short-lived effector cells during infection and are subsequently enriched for a CM phenotype during the memory phase [45]. This effector vs. memory divergence is further reflected by CCR2 expression, where CCR2^+^ cells are better poised toward an effector fate, while CCR2^−^ cells serve as memory precursors which further could acquire the tissue-residency phenotype following infection [47]. Consistent with this framework, our single-cell-based analysis suggests that heterogeneity also exists within aged VM CD8^+^ T cell subsets and that Ccr2 expression may be associated with cytotoxic VM CD8^+^ T cells during aging (Figs. 1 and 2). However, further validation experiments are required to define the extent and functional significance of heterogeneity within aged VM subsets.

Adipose tissue is considered one of the key niches for understanding age-related immune remodeling [123], functioning both as a reservoir for memory T cells [124–126] and as a site of age-associated immune cell accumulation [42, 127–129]. Importantly, CD8^+^ T cells have been associated with aging and inflammation [13, 14, 42, 128, 130, 131], making it pertinent to dissect the heterogeneity within VM pools and identify the subsets that drive inflammation during aging. Recent single-cell RNA sequencing combined with RNA velocity and trajectory analyses of CD8⁺ T cells from aged adipose tissue revealed that VM CD8⁺ T cells in this compartment are transcriptionally heterogeneous [42] (Figs. 1 and 2). Trajectory analyses identified a differentiation continuum in which cytotoxic-like VM CD8^+^ T cells, expressing granzyme M, arise from the broader VM pool. These effectors subsequently transition into a quiescent state with reduced translational and metabolic activity but sustained *Bcl2* expression and cytokine receptor gene expression, consistent with the survival adaptations as described earlier.

While the effector function of VM CD8^+^ T cells could be beneficial to the aged host during infection and provide protection against infections [43], their chronic accumulation could be pathological. CD8⁺ T cells, including cytotoxic VM pools have been implicated in granzyme-mediated tissue inflammation in aged hosts [14, 42, 132, 133]. This pathological potential is further supported by the observation that granzyme B-expressing innate cytotoxic VM CD8^+^ T cell subset, differentiated from VM pools, drive pathological condition alopecia areata in an NKG2D-dependent manner, and NKG2D blockade is sufficient to attenuate disease [48]. These findings underscore the need to identify other regulatory proteins expressed on VM CD8^+^ T cells during aging that could have functional relevance.

#### Inhibitory receptors on VM CD8^+^ T cells

Trajectory analyses of CD8^+^ T cells from aged adipose tissue identified the inhibitory receptor *Fcgr2b* as one of the notable genes expressed in cytotoxic-like VM CD8^+^ T cell subset [42] (Fig. 2). FcγRIIb is an inhibitory receptor expressed on B cells that limits the BCR signaling [134]. Interestingly, the presence of B cell inhibitory receptors on VM CD8^+^ T cells is not confined to FcγRIIb. Elevated expression of CD22, another inhibitory receptor expressed on B cells [135], has been reported on IL-4-driven VM CD8⁺ T cell subsets with an activated phenotype expressing granzyme A, granzyme M, and IFN-γ [46]. CD22 functions as an intrinsic negative regulator of the IL-4-induced effector program, limiting the expansion and responsiveness of VM CD8^+^ T cells during *Heligmosomoides polygyrus* infection [46]. Together, these suggest that the functional output of VM CD8^+^ T cells may be influenced by inhibitory receptors classically known to express on B cells.

Earlier, FcγRIIb has been shown to be expressed on memory CD8^+^ T cells and on activated CD8^+^ T cells in tumor and infection settings, where it regulates memory cell expansion and mediates apoptosis in an Fgl2-dependent manner [136–140]. During aging, elevated *Fcgr2b* expression has also been reported on regulatory CD8^+^ T cells, however, its functional relevance has not been determined [141]. Interestingly, IgG, a ligand for FcγRIIb, accumulates with age and has been implicated as driver of aging [142, 143]. Considered together with trajectory analyses of aged VM CD8^+^ T cells, these observations suggest that *Fcgr2b* activation may regulate the innate-like cytotoxic VM CD8^+^ T cell pool via IgG-mediated apoptosis (Fig. 1). Notably, these *Fcgr2b*-expressing innate-like cytotoxic VM CD8^+^ T cells were found to express *Avcr2a*, *Klra3*,* Klrc1*, *Klrk1*, *Ctla2a*,* Stat1*,* Runx3*, suggesting a possible regulatory role of these cells during aging [42] (Figs. 1 and 2).

Supporting this, evidence suggests that elevated *Fcgr2b* expression is part of a distinct age-associated VM CD8^+^ T cell signature and that FcγRIIB-expressing aged VM cells may be prone to apoptosis [144]. These observations are compatible with the idea that Fcgr2b may influence the emergence or regulation of a cytotoxic/inflammatory VM state. At the same time, blockade of FcγRIIB has been suggested to enhance CD8^+^ T cell responses [144], further raising the possibility that FcγRIIb contributes to age-associated dysfunction in VM CD8^+^ T cells. However, further mechanistic studies are required to determine whether FcγRIIb directly drives cytotoxic differentiation, mediates IgG-dependent apoptotic control, or instead marks a terminal or regulatory state within the aged VM compartment.

### VM-like CD8^+^ T cells in human aging and inflammation

The VM phenotype has been most clearly defined in mice, whereas defining an equivalent antigen-inexperienced VM CD8⁺ T cell population in humans remains more challenging, cause of phenotypic changes induced by infection history. A human VM-like CD8^+^ T cells has been described within a CD45RA^+^ CD27^−^ compartment [48, 107, 145, 146]. However, the phenotypic markers used to define naïve, memory, TEMRA, and VM CD8^+^ T cells are highly overlapping [117, 147, 148]. While CD45RA was historically considered an exclusive marker of naïve T cells, its expression can also be induced on memory CD8⁺ T cells during CMV infection [147]. As discussed earlier, infections such as CMV induce phenotypic changes within the CD8⁺ T cell compartment, including loss of CD27 [115, 118, 119], and therefore require additional caution when defining human VM CD8⁺ T cells.

However, these putative human VM cells express low levels of CD5 [36] and express CD122, Nur77, and EOMES, consistent with IL-15 responsiveness and elevated basal TCR signaling, two central features of murine VM CD8⁺ T cells [25–27, 36]. They also express the inhibitory receptors KIR (killer-cell immunoglobulin-like receptor) and NKG2A, show loss of CCR7 and CD27, and have therefore been proposed as a close human VM-like counterpart [25–27, 36, 52, 115, 117–119, 146, 149]. Functionally, these human CD45RA^+^ Eomes^+^ KIR / NKG2A^+^ T cells resemble murine VM CD8^+^ T cells in their ability to mount effector-like responses and cytotoxicity following IL-12 and IL-18 stimulation [36, 146]. Interestingly, these cells accumulate with age in blood and spleen, and exhibited localization in liver and skin, like murine VM CD8^+^ T cells [36, 48, 107, 145]. Like murine VM cells, human VM-like CD8^+^ T cells exhibit proliferation defects following TCR stimulation and display high spare respiratory capacity as discussed previously, and have also been linked to inflammatory disease, including alopecia areata [48, 107, 109].

Interestingly, human CD62L⁺CD45RO⁺CD25⁺ memory-like CD8⁺ T cells with a highly diverse TCR repertoire have been shown to provide protection against influenza virus [150, 151] and may compensate for the loss of naïve CD8⁺ T cells in aged individuals [152]. Similar findings were reported for VM CD8⁺ T cells in aged mice by Lanzer et al. [43], although it remains unclear whether this CD62L⁺CD45RO⁺CD25⁺ subset truly represents a population of VM-like CD8⁺ T cells in humans. That said, whether human VM-like CD8^+^ T cells simply accumulate with age or are shaped by infection history and whether they compensate for naïve CD8⁺ T cell loss or contribute to inflammation in humans requires further investigation.

Finally, the contribution of VM-like CD8⁺ T cells to inflammaging must be considered in the context of multiple inflammatory drivers, including senescent cells with a senescence-associated secretory phenotype (SASP), aged innate immune cells, adipose tissue inflammation, persistent infection, and cytokines such as IL-6, IL-1β, and TNF-α.

### Conclusion and open questions

Functional and phenotypic heterogeneity has emerged as a defining feature of the VM CD8⁺ T cell compartment during aging. Current evidence supports a model in which one fraction of aged VM CD8⁺ T cells retain memory-like properties, whereas another acquires innate-like cytotoxic characteristics. Although aged VM CD8⁺ T cells show impaired proliferation following TCR stimulation, they remain highly responsive to homeostatic cytokines and can mount rapid effector-like responses. In the context of age-associated loss of naïve CD8⁺ T cells, these properties may help preserve early immune protection. At the same time, persistent accumulation of cytotoxic VM subsets may also have pathological consequences, particularly if sustained granzyme expression contributes to chronic tissue inflammation in aged hosts.

A major unresolved issue is therefore whether cytotoxic VM CD8⁺ T cells are primarily protective, pathogenic, or context-dependent. Addressing this question will require a better understanding of the heterogeneity of VM CD8⁺ T cell subsets across tissues, the signals that govern their differentiation, and their fate following infection in aged hosts (Fig. 1). It will also be important to determine whether distinct tissue environments selectively promote either memory-like or cytotoxic VM states.

A second set of open questions concerns the regulation of aged VM CD8⁺ T cells by inhibitory receptors, particularly Fcgr2b. Although Fcgr2b expression has been observed in aged VM-associated cytotoxic subsets, the timing, anatomical distribution, and functional significance of this expression remain unclear. It is not yet known which host-derived signals induce *Fcgr2b* expression in VM CD8⁺ T cells, whether FcγRIIb⁺ and FcγRIIb⁻ VM subsets differ in survival or effector capacity, or whether FcγRIIb preferentially marks a terminal, regulatory, or apoptosis-prone cytotoxic state. These questions are especially intriguing considering age-associated IgG accumulation, which raises the possibility that IgG- FcγRIIb interactions may shape the fate, cytotoxicity, or inflammatory potential of VM CD8⁺ T cells (Fig. 1). However, this possibility requires direct functional testing.

Finally, the relevance of VM CD8⁺ T cells to human aging remains insufficiently defined. Human VM-like CD8⁺ T cell populations share several phenotypic and functional features with murine VM cells, but whether they play comparable protective or inflammatory roles during aging is still unclear. Lifelong infection history, including persistent infections such as CMV, as well as repeated acute inflammatory exposures, may substantially remodel this compartment in humans. Resolving these questions will require integrated single-cell, spatial, and functional studies in mice together with careful phenotypic characterization in human aging cohorts. Such work should clarify whether VM CD8⁺ T cells act mainly as compensatory effectors in the aging immune system or whether they also represent a driver of age-associated inflammatory disease.

## Data Availability

The single-cell RNA-sequencing dataset used for Figure 2 was reported previously [42] and is openly available in the Gene Expression Omnibus (GEO) under accession number GSE300036.

## Abbreviations

AIMT: antigen-inexperienced memory T cells
AP-1: activator protein 1
BATF3: basic leucine zipper transcription factor ATF-like 3
BCR: B-cell receptor
CD: cluster of differentiation
CM: central memory
CMV: cytomegalovirus
CXCR3: C-X-C motif chemokine receptor 3
DCs: dendritic cells
EM: effector memory
EOMES: eomesodermin
ERK1/2: extracellular signal-regulated kinase 1/2
Fcgr2b: Fc gamma receptor IIb
HP: homeostatic proliferation
IFN-γ: interferon gamma
IgG: immunoglobulin G
IL: interleukin
IM: innate memory
ITK: IL-2-inducible T cell kinase
KIR: killer-cell immunoglobulin-like receptor
KLRG1: killer cell lectin-like receptor subfamily G member 1
LCMV: lymphocytic choriomeningitis virus
LM: Listeria monocytogenes
MAPK: mitogen-activated protein kinase
NKG2A: natural killer group 2 A
NKG2D: natural killer group 2D
NKT: natural killer T cells
SP: single positive
STAT4: signal transducer and activator of transcription 4
TCR: T cell receptor
TNF-α: tumor necrosis factor alpha
VM: virtual memory

## References

[1] Niccoli T, Partridge L. Ageing as a risk factor for disease. Curr Biol. 2012;22(17):R741–752. 10.1016/j.cub.2012. 07.024 PubMed PMID: 22975005. 22975005

[2] Montecino-Rodriguez E, Berent-Maoz B, Dorshkind K. Causes, consequences, and reversal of immune system aging. J Clin Invest. 2013;123(3):958–65. doi:10.1172/JCI64096 PubMed PMID: 23454758; PubMed Central PMCID: PMC3582124. 10.1172/JCI64096 PMC3582124 23454758

[3] López-Otín C, Blasco MA, Partridge L, Serrano M, Kroemer G. Hallmarks of aging: An expanding universe. Cell. 2023;186(2):243–78. 10.1016/j.cell.2022.11. .001 PubMed PMID: 36599349. 36599349

[4] Fang M, Sigal LJ. Antibodies and CD8 + T cells are complementary and essential for natural resistance to a highly lethal cytopathic virus. J Immunol. 2005;175(10):6829–36. 10.4049/jimmunol.175.10.6829 . PubMed PMID: 16272340. 16272340

[5] Koh CH, Lee S, Kwak M, Kim BS, Chung Y. CD8 T-cell subsets: heterogeneity, functions, and therapeutic potential. Exp Mol Med. 2023;55(11):2287–99. 10.1038/s12276-023-01105-x . PubMed PMID: 37907738; PubMed Central PMCID: PMC10689838. PMC10689838 37907738

[6] Karupiah G, Buller RM, Van Rooijen N, Duarte CJ, Chen J. Different roles for CD4 + and CD8 + T lymphocytes and macrophage subsets in the control of a generalized virus infection. J Virol. 1996;70(12):8301–9. 10.1128/JVI.70.12.8301-8309. 1996 PubMed PMID: 8970949; PubMed Central PMCID: PMC190917. PMC190917 8970949

[7] Carrasco E, Gómez de Las Heras MM, Gabandé-Rodríguez E, Desdín-Micó G, Aranda JF, Mittelbrunn M. The role of T cells in age-related diseases. Nat Rev Immunol. 2022;22(2):97–111. 10.1038/s41577-021-00557-4 . PubMed PMID: 34099898. 34099898

[8] Liang Z, Dong X, Zhang Z, Zhang Q, Zhao Y. Age-related thymic involution: Mechanisms and functional impact. Aging Cell. 2022;21(8):e13671. 10.1111/acel.13671 . PubMed PMID: 35822239; PubMed Central PMCID: PMC9381902. PMC9381902 35822239

[9] Thomas R, Wang W, Su DM. Contributions of Age-Related Thymic Involution to Immunosenescence and Inflammaging. Immun Ageing. 2020;17:2. 10.1186/s12979. 020-0173-8 PubMed PMID: 31988649; PubMed Central PMCID: PMC6971920. PMC6971920 31988649

[10] Briceño O, Lissina A, Wanke K, Afonso G, von Braun A, Ragon K, et al. Reduced naïve CD8(+) T-cell priming efficacy in elderly adults. Aging Cell. 2016;15(1):14–21. 10.1111/acel.12384 . PubMed PMID: 26472076; PubMed Central PMCID: PMC4717282. PMC4717282 26472076

[11] Lee JB, Oelke M, Ramachandra L, Canaday DH, Schneck JP. Decline of influenza-specific CD8 + T cell repertoire in healthy geriatric donors. Immun Ageing. 2011;8:6. 10.1186/1742-4933-8-6 . PubMed PMID: 21846352; PubMed Central PMCID: PMC3179433. PMC3179433 21846352

[12] Yager EJ, Ahmed M, Lanzer K, Randall TD, Woodland DL, Blackman MA. Age-associated decline in T cell repertoire diversity leads to holes in the repertoire and impaired immunity to influenza virus. J Exp Med. 2008;205(3):711–23. 10.1084/jem.20071140 . PubMed PMID: 18332179; PubMed Central PMCID: PMC2275391. PMC2275391 18332179

[13] Mittelbrunn M, Kroemer G. Hallmarks of T cell aging. Nat Immunol. 2021;22(6):687–98. 10.1038/s41590-021. 00927-z PubMed PMID: 33986548. 33986548

[14] Mogilenko DA, Shpynov O, Andhey PS, Arthur L, Swain A, Esaulova E, et al. Comprehensive Profiling of an Aging Immune System Reveals Clonal GZMK+ CD8 + T Cells as Conserved Hallmark of Inflammaging. Immunity. 2021;54(1):99–e11512. 10.1016/j.immuni.2020.11.005 . PubMed PMID: 33271118. 33271118

[15] Ferrucci L, Fabbri E. Inflammageing: chronic inflammation in ageing, cardiovascular disease, and frailty. Nat Rev Cardiol. 2018;15(9):505–22. 10.1038/s41569-018-0064-2 . PubMed PMID: 30065258; PubMed Central PMCID: PMC6146930. PMC6146930 30065258

[16] Turner SJ, Bennett TJ, La Gruta NL. CD8 + T-Cell Memory: The Why, the When, and the How. Cold Spring Harb Perspect Biol. 2021;13(5):a038661. 10.1101/cshperspect.a038661 . PubMed PMID: 33648987; PubMed Central PMCID: PMC8091951. PMC8091951 33648987

[17] Jameson SC. The Naming of Memory T-Cell Subsets. Cold Spring Harb Perspect Biol. 2021;13(1):a037788. 10.1101. /cshperspect.a037788 PubMed PMID: 33229439; PubMed Central PMCID: PMC7778151. 10.1101/cshperspect.a037788 PMC7778151 33229439

[18] Martin MD, Badovinac VP. Defining Memory CD8 T Cell. Front Immunol. 2018;9:2692. 10.3389/fimmu. 2018.02692 PubMed PMID: 30515169; PubMed Central PMCID: PMC6255921. PMC6255921 30515169

[19] Mueller SN, Gebhardt T, Carbone FR, Heath WR. Memory T cell subsets, migration patterns, and tissue residence. Annu Rev Immunol. 2013;31:137–61. 10.1146/annurev-immunol-032712. -095954 PubMed PMID: 23215646. 23215646

[20] Samji T, Khanna KM. Understanding memory CD8 + T cells. Immunol Lett. 2017;185:32–9. 10.1016/j.imlet. 2017.02.012 PubMed PMID: 28274794; PubMed Central PMCID: PMC5508124. PMC5508124 28274794

[21] Nolz JC, Starbeck-Miller GR, Harty JT. Naive, effector and memory CD8 T-cell trafficking: parallels and distinctions. Immunotherapy. 2011;3(10):1223–33. 10.2217/imt.11.100 . PubMed PMID: 21995573; PubMed Central PMCID: PMC3214994. PMC3214994 21995573

[22] Butler NS, Nolz JC, Harty JT. Immunologic considerations for generating memory CD8 T cells through vaccination. Cell Microbiol. 2011;13(7):925–33. 10.1111/j.1462-5822.2011.01594. x PubMed PMID: 21501363; PubMed Central PMCID: PMC3116979. PMC3116979 21501363

[23] Wherry EJ, Ahmed R. Memory CD8 T-cell differentiation during viral infection. J Virol. 2004;78(11):5535–45. 10.1128/JVI.78.11.5535-5545. 2004 PubMed PMID: 15140950; PubMed Central PMCID: PMC415833. PMC415833 15140950

[24] Masopust D, Vezys V, Marzo AL, Lefrançois L. Preferential localization of effector memory cells in nonlymphoid tissue. Science. 2001;291(5512):2413–7. 10.1126/science.1058867 . PubMed PMID: 11264538. 11264538

[25] Yang B, Dewals BG. Going Virtual as a Memory-Phenotype T Lymphocyte. Eur J Immunol. 2026;56(3):e70174. 10.1002/eji.70174 . PubMed PMID: 41838023. 41838023

[26] White JT, Cross EW, Kedl RM. Antigen-inexperienced memory CD8 + T cells: where they come from and why we need them. Nat Rev Immunol. 2017;17(6):391–400. 10.1038/nri.2017.34 . PubMed PMID: 28480897; PubMed Central PMCID: PMC5569888. PMC5569888 28480897

[27] Hussain T, Quinn KM. Similar but different: virtual memory CD8 T cells as a memory-like cell population. Immunol Cell Biol. 2019;97(7):675–84. 10.1111/imcb.12277 . PubMed PMID: 31140625. 31140625

[28] Lee YJ, Holzapfel KL, Zhu J, Jameson SC, Hogquist KA. Steady-state production of IL-4 modulates immunity in mouse strains and is determined by lineage diversity of iNKT cells. Nat Immunol. 2013;14(11):1146–54. 10.1038/ni.2731 . PubMed PMID: 24097110; PubMed Central PMCID: PMC3824254. PMC3824254 24097110

[29] Lai D, Zhu J, Wang T, Hu-Li J, Terabe M, Berzofsky JA, et al. KLF13 sustains thymic memory-like CD8(+) T cells in BALB/c mice by regulating IL-4-generating invariant natural killer T cells. J Exp Med. 2011;208(5):1093–103. 10.1084/jem.20101527 . PubMed PMID: 21482696; PubMed Central PMCID: PMC3092346. PMC3092346 21482696

[30] Alonzo ES, Sant’Angelo DB. Development of PLZF-expressing innate T cells. Curr Opin Immunol. 2011;23(2):220–7. 10.1016/j.coi. .2010.12.016 PubMed PMID: 21257299; PubMed Central PMCID: PMC3073746. PMC3073746 21257299

[31] Dubois S, Waldmann TA, Müller JR. ITK and IL-15 support two distinct subsets of CD8 + T cells. Proc Natl Acad Sci U S A. 2006;103(32):12075–80. 10.1073/pnas.0605212103 . PubMed PMID: 16880398; PubMed Central PMCID: PMC1567699. PMC1567699 16880398

[32] Hu J, Sahu N, Walsh E, August A. Memory phenotype CD8 + T cells with innate function selectively develop in the absence of active Itk. Eur J Immunol. 2007;37(10):2892–9. 10.1002/eji.200737311 . PubMed PMID: 17724684; PubMed Central PMCID: PMC2770953. PMC2770953 17724684

[33] Atherly LO, Lucas JA, Felices M, Yin CC, Reiner SL, Berg LJ. The Tec family tyrosine kinases Itk and Rlk regulate the development of conventional CD8 + T cells. Immunity. 2006;25(1):79–91. 10.1016/j.immuni.2006.05.012 . PubMed PMID: 16860759. 16860759

[34] Broussard C, Fleischacker C, Horai R, Chetana M, Venegas AM, Sharp LL, et al. Altered development of CD8 + T cell lineages in mice deficient for the Tec kinases Itk and Rlk. Immunity. 2006;25(1):93–104. 10.1016/j.immuni.2006.05.011 . PubMed PMID: 16860760. 16860760

[35] Drobek A, Moudra A, Mueller D, Huranova M, Horkova V, Pribikova M, et al. Strong homeostatic TCR signals induce formation of self-tolerant virtual memory CD8 T cells. EMBO J. 2018;37(14):e98518. 10.15252/embj.201798518 . PubMed PMID: 29752423; PubMed Central PMCID: PMC6043851. PMC6043851 29752423

[36] White JT, Cross EW, Burchill MA, Danhorn T, McCarter MD, Rosen HR, et al. Virtual memory T cells develop and mediate bystander protective immunity in an IL-15-dependent manner. Nat Commun. 2016;7:11291. 10.1038/ncomms11291 . PubMed PMID: 27097762; PubMed Central PMCID: PMC4844673. PMC4844673 27097762

[37] Sosinowski T, White JT, Cross EW, Haluszczak C, Marrack P, Gapin L, et al. CD8α + dendritic cell trans presentation of IL-15 to naive CD8 + T cells produces antigen-inexperienced T cells in the periphery with memory phenotype and function. J Immunol. 2013;190(5):1936–47. 10.4049/jimmunol.1203149 . PubMed PMID: 23355737; PubMed Central PMCID: PMC3578102. PMC3578102 23355737

[38] Akue AD, Lee JY, Jameson SC. Derivation and maintenance of virtual memory CD8 T cells. J Immunol. 2012;188(6):2516–23. 10.4049/jimmunol.1102213 . PubMed PMID: 22308307; PubMed Central PMCID: PMC3294185. PMC3294185 22308307

[39] Haluszczak C, Akue AD, Hamilton SE, Johnson LDS, Pujanauski L, Teodorovic L, et al. The antigen-specific CD8 + T cell repertoire in unimmunized mice includes memory phenotype cells bearing markers of homeostatic expansion. J Exp Med. 2009;206(2):435–48. 10.1084/jem.20081829 . PubMed PMID: 19188498; PubMed Central PMCID: PMC2646575. PMC2646575 19188498

[40] Lee YJ, Jameson SC, Hogquist KA. Alternative memory in the CD8 T cell lineage. Trends Immunol. 2011;32(2):50–6. 10.1016/j.it. .2010.12.004 PubMed PMID: 21288770; PubMed Central PMCID: PMC3039080. PMC3039080 21288770

[41] Jameson SC, Lee YJ, Hogquist KA. Innate memory T cells. Adv Immunol. 2015;126:173–213. 10.1016/bs.ai. .2014.12.001 PubMed PMID: 25727290; PubMed Central PMCID: PMC4670568. PMC4670568 25727290

[42] Kumar A, O’Brien M, Young VB, Yung R, Single-Cell RNA. Sequencing Identifies Accumulation of Fcgr2b + Virtual Memory-Like CD8 T Cells With Cytotoxic and Inflammatory Potential in Aged Mouse White Adipose Tissue. Aging Cell. 2025;24(12):e70278. 10.1111/acel.70278 . PubMed PMID: 41116748; PubMed Central PMCID: PMC12686556. PMC12686556 41116748

[43] Lanzer KG, Cookenham T, Reiley WW, Blackman MA. Virtual memory cells make a major contribution to the response of aged influenza-naïve mice to influenza virus infection. Immun Ageing. 2018;15:17. 10.1186/s12979-018-0122-y . PubMed PMID: 30093911; PubMed Central PMCID: PMC6081820. PMC6081820 30093911

[44] Chu T, Tyznik AJ, Roepke S, Berkley AM, Woodward-Davis A, Pattacini L, et al. Bystander-activated memory CD8 T cells control early pathogen load in an innate-like, NKG2D-dependent manner. Cell Rep. 2013;3(3):701–8. .020 PubMed PMID: 23523350; PubMed Central PMCID: PMC3628815. 10.1016/j.celrep.2013.02.020 PMC3628815 23523350

[45] Lee JY, Hamilton SE, Akue AD, Hogquist KA, Jameson SC. Virtual memory CD8 T cells display unique functional properties. Proc Natl Acad Sci U S A. 2013;110(33):13498–503. 10.1073/pnas.1307572110 . PubMed PMID: 23898211; PubMed Central PMCID: PMC3746847. PMC3746847 23898211

[46] Yang B, Piedfort O, Sanchez-Sanchez G, Lavergne A, Gong M, Peng G, et al. IL-4 induces CD22 expression to restrain the effector program of virtual memory T cells. Sci Immunol. 2025;10(104):eadk4841. 10.1126/sciimmunol.adk. 4841 PubMed PMID: 39919198. 39919198

[47] Hou S, Shao T, Mao T, Shi J, Sun J, Mei M, et al. Virtual memory T cells orchestrate extralymphoid responses conducive to resident memory. Sci Immunol. 2021;6(62):eabg9433. 10.1126/sciimmunol.abg. 9433 PubMed PMID: 34855456. 34855456

[48] Seok J, Cho SD, Lee J, Choi Y, Kim SY, Lee SM, et al. A virtual memory CD8 + T cell-originated subset causes alopecia areata through innate-like cytotoxicity. Nat Immunol. 2023;24(8):1308–17. 10.1038/s41590-023-01547-5 . PubMed PMID: 37365384. 37365384

[49] Sallusto F, Geginat J, Lanzavecchia A. Central memory and effector memory T cell subsets: function, generation, and maintenance. Annu Rev Immunol. 2004;22:745–63. 10.1146/annurev.immunol.22.012703.104702 . PubMed PMID: 15032595. 15032595

[50] Sallusto F, Lenig D, Förster R, Lipp M, Lanzavecchia A. Two subsets of memory T lymphocytes with distinct homing potentials and effector functions. Nature. 1999;401(6754):708–12. 10. 1038/44385 PubMed PMID: 10537110. 10.1038/44385 10537110

[51] Kaech SM, Ahmed R. Memory CD8 + T cell differentiation: initial antigen encounter triggers a developmental program in naïve cells. Nat Immunol. 2001;2(5):415–22. doi:10.1038/87720 PubMed PMID: 11323695; PubMed Central PMCID: PMC3760150. 10.1038/87720 PMC3760150 11323695

[52] Jarjour NN, Dalzell TS, Maurice NJ, Wanhainen KM, Peng C, O’Flanagan SD, et al. Collaboration between interleukin-7 and – 15 enables adaptation of tissue-resident and circulating memory CD8 + T cells to cytokine deficiency. Immunity. 2025;58(3):616–e6315. 10.1016/j.immuni.2025.02. .009 PubMed PMID: 40023156. PMC13329436 40023156

[53] Jung YW, Kim HG, Perry CJ, Kaech SM. CCR7 expression alters memory CD8 T-cell homeostasis by regulating occupancy in IL-7- and IL-15-dependent niches. Proc Natl Acad Sci U S A. 2016;113(29):8278–83. 10.1073/pnas.1602899113 . PubMed PMID: 27385825; PubMed Central PMCID: PMC4961181. PMC4961181 27385825

[54] Ikuta K, Hara T, Abe S, Asahi T, Takami D, Cui G. The Roles of IL-7 and IL-15 in Niches for Lymphocyte Progenitors and Immune Cells in Lymphoid Organs. Curr Top Microbiol Immunol. 2021;434:83–101. 10.1007/978-3. -030-86016-5_4 PubMed PMID: 34850283. 34850283

[55] Chiu BC, Martin BE, Stolberg VR, Chensue SW. Cutting edge: Central memory CD8 T cells in aged mice are virtual memory cells. J Immunol. 2013;191(12):5793–6. 10.4049/jimmunol.1302509 . PubMed PMID: 24227783; PubMed Central PMCID: PMC3858473. PMC3858473 24227783

[56] Weinreich MA, Odumade OA, Jameson SC, Hogquist KA. T cells expressing the transcription factor PLZF regulate the development of memory-like CD8 + T cells. Nat Immunol. 2010;11(8):709–16. 10.1038/ni.1898 . PubMed PMID: 20601952; PubMed Central PMCID: PMC3051359. PMC3051359 20601952

[57] Weinreich MA, Takada K, Skon C, Reiner SL, Jameson SC, Hogquist KA. KLF2 transcription-factor deficiency in T cells results in unrestrained cytokine production and upregulation of bystander chemokine receptors. Immunity. 2009;31(1):122–30. 10.1016/j.immuni.2009.05.011 . PubMed PMID: 19592277; PubMed Central PMCID: PMC2724594. PMC2724594 19592277

[58] Berton RR, Heidarian M, Kannan SK, Shah M, Butler NS, Harty JT, et al. Accurate enumeration of pathogen-specific and virtual memory CD8 T cells after infection. J Immunol. 2025;214(5):995–1007. 10.1093/jimmun/vkaf007 . PubMed PMID: 40167212; PubMed Central PMCID: PMC12123210. PMC12123210 40167212

[59] Hogquist KA. Signal strength in thymic selection and lineage commitment. Curr Opin Immunol. 2001;13(2):225–31. 10.1016/s0952-7915. (00)00208-9 PubMed PMID: 11228417. 11228417

[60] Klein L, Kyewski B, Allen PM, Hogquist KA. Positive and negative selection of the T cell repertoire: what thymocytes see (and don’t see). Nat Rev Immunol. 2014;14(6):377–91. 10.1038/nri3667 . PubMed PMID: 24830344; PubMed Central PMCID: PMC4757912. PMC4757912 24830344

[61] Punt JA, Suzuki H, Granger LG, Sharrow SO, Singer A. Lineage commitment in the thymus: only the most differentiated (TCRhibcl-2hi) subset of CD4 + CD8+ thymocytes has selectively terminated CD4 or CD8 synthesis. J Exp Med. 1996;184(6):2091–9. .2091 PubMed PMID: 8976166; PubMed Central PMCID: PMC2196385. 10.1084/jem.184.6.2091 PMC2196385 8976166

[62] Nitta T, Nitta S, Lei Y, Lipp M, Takahama Y. CCR7-mediated migration of developing thymocytes to the medulla is essential for negative selection to tissue-restricted antigens. Proc Natl Acad Sci U S A. 2009;106(40):17129–33. 10.1073/pnas.0906956106 . PubMed PMID: 19805112; PubMed Central PMCID: PMC2761327. PMC2761327 19805112

[63] Xu X, Zhang S, Li P, Lu J, Xuan Q, Ge Q. Maturation and emigration of single-positive thymocytes. Clin Dev Immunol. 2013;2013:282870. 10.1155/2013/282870 . PubMed PMID: 24187562; PubMed Central PMCID: PMC3804360. PMC3804360 24187562

[64] Berard M, Tough DF. Qualitative differences between naïve and memory T cells. Immunology. 2002;106(2):127–38. 10.1046/j.1365-2567. 2002.01447.x PubMed PMID: 12047742; PubMed Central PMCID: PMC1782715. PMC1782715 12047742

[65] Weinreich MA, Hogquist KA. Thymic emigration: when and how T cells leave home. J Immunol. 2008;181(4):2265–70. 10.4049/jimmunol.181. .4.2265 PubMed PMID: 18684914; PubMed Central PMCID: PMC2861282. PMC2861282 18684914

[66] Banerjee A, Gordon SM, Intlekofer AM, Paley MA, Mooney EC, Lindsten T, et al. Cutting edge: The transcription factor eomesodermin enables CD8 + T cells to compete for the memory cell niche. J Immunol. 2010;185(9):4988–92. 10.4049/jimmunol.1002042 . PubMed PMID: 20935204; PubMed Central PMCID: PMC2975552. PMC2975552 20935204

[67] McLane LM, Banerjee PP, Cosma GL, Makedonas G, Wherry EJ, Orange JS, et al. Differential localization of T-bet and Eomes in CD8 T cell memory populations. J Immunol. 2013;190(7):3207–15. 10.4049/jimmunol.1201556 . PubMed PMID: 23455505; PubMed Central PMCID: PMC3608800. PMC3608800 23455505

[68] Kedl RM, White JT. Foreign antigen-independent memory-phenotype CD4 + T cells: a new player in innate immunity? Nat Rev Immunol. 2018;18(3):1. 10.1038/nri.2018.13 . PubMed PMID: 29480288; PubMed Central PMCID: PMC6191184. PMC6191184 29480288

[69] Viano ME, Baez NS, Savid-Frontera C, Lidon NL, Hodge DL, Herbelin A, et al. Virtual Memory CD8 + T Cells: Origin and Beyond. J Interferon Cytokine Res. 2022;42(12):624–42. 10.1089/jir.2022.0053 . PubMed PMID: 36083273; PubMed Central PMCID: PMC9835308. PMC9835308 36083273

[70] Lee YJ, Wang H, Starrett GJ, Phuong V, Jameson SC, Hogquist KA. Tissue-Specific Distribution of iNKT Cells Impacts Their Cytokine Response. Immunity. 2015;43(3):566–78. 10.1016/j.immuni.2015.06.025 . PubMed PMID: 26362265; PubMed Central PMCID: PMC4575275. PMC4575275 26362265

[71] Kurzweil V, LaRoche A, Oliver PM. Increased peripheral IL-4 leads to an expanded virtual memory CD8 + population. J Immunol. 2014;192(12):5643–51. 10.4049/jimmunol.1301755 . PubMed PMID: 24795452; PubMed Central PMCID: PMC4097049. PMC4097049 24795452

[72] Martinet V, Tonon S, Torres D, Azouz A, Nguyen M, Kohler A, et al. Type I interferons regulate eomesodermin expression and the development of unconventional memory CD8(+) T cells. Nat Commun. 2015;6:7089. 10.1038/ncomms8089 . PubMed PMID: 25953241; PubMed Central PMCID: PMC4432629. PMC4432629 25953241

[73] Tan JT, Dudl E, LeRoy E, Murray R, Sprent J, Weinberg KI, et al. IL-7 is critical for homeostatic proliferation and survival of naive T cells. Proc Natl Acad Sci U S A. 2001;98(15):8732–7. 10.1073/pnas.161126098 . PubMed PMID: 11447288; PubMed Central PMCID: PMC37504. PMC37504 11447288

[74] Cho JH, Boyman O, Kim HO, Hahm B, Rubinstein MP, Ramsey C, et al. An intense form of homeostatic proliferation of naive CD8 + cells driven by IL-2. J Exp Med. 2007;204(8):1787–801. 10.1084/jem.20070740 . PubMed PMID: 17664294; PubMed Central PMCID: PMC2118670. PMC2118670 17664294

[75] Goldrath AW, Sivakumar PV, Glaccum M, Kennedy MK, Bevan MJ, Benoist C, et al. Cytokine requirements for acute and Basal homeostatic proliferation of naive and memory CD8 + T cells. J Exp Med. 2002;195(12):1515–22. 10.1084/jem.20020033 . PubMed PMID: 12070279; PubMed Central PMCID: PMC2193554. PMC2193554 12070279

[76] Sandau MM, Winstead CJ, Jameson SC. IL-15 is required for sustained lymphopenia-driven proliferation and accumulation of CD8 T cells. J Immunol. 2007;179(1):120–5. 10.4049/jimmunol.179.1.120 . PubMed PMID: 17579029. 17579029

[77] Tan JT, Ernst B, Kieper WC, LeRoy E, Sprent J, Surh CD. Interleukin (IL)-15 and IL-7 jointly regulate homeostatic proliferation of memory phenotype CD8 + cells but are not required for memory phenotype CD4 + cells. J Exp Med. 2002;195(12):1523–32. 10.1084/jem.20020066 . PubMed PMID: 12070280; PubMed Central PMCID: PMC2193564. PMC2193564 12070280

[78] Joshi NS, Cui W, Chandele A, Lee HK, Urso DR, Hagman J, et al. Inflammation directs memory precursor and short-lived effector CD8(+) T cell fates via the graded expression of T-bet transcription factor. Immunity. 2007;27(2):281–95. 10.1016/j.immuni.2007.07.010 . PubMed PMID: 17723218; PubMed Central PMCID: PMC2034442. PMC2034442 17723218

[79] Intlekofer AM, Takemoto N, Kao C, Banerjee A, Schambach F, Northrop JK, et al. Requirement for T-bet in the aberrant differentiation of unhelped memory CD8 + T cells. J Exp Med. 2007;204(9):2015–21. 10.1084/jem.20070841 . PubMed PMID: 17698591; PubMed Central PMCID: PMC2118697. PMC2118697 17698591

[80] Intlekofer AM, Takemoto N, Wherry EJ, Longworth SA, Northrup JT, Palanivel VR, et al. Effector and memory CD8 + T cell fate coupled by T-bet and eomesodermin. Nat Immunol. 2005;6(12):1236–44. 10.1038/ni1268 . PubMed PMID: 16273099. 16273099

[81] Rao RR, Li Q, Odunsi K, Shrikant PA. The mTOR kinase determines effector versus memory CD8 + T cell fate by regulating the expression of transcription factors T-bet and Eomesodermin. Immunity. 2010;32(1):67–78. 10.1016/j.immuni.2009.10.010 . PubMed PMID: 20060330; PubMed Central PMCID: PMC5836496. PMC5836496 20060330

[82] Gordon SM, Carty SA, Kim JS, Zou T, Smith-Garvin J, Alonzo ES, et al. Requirements for eomesodermin and promyelocytic leukemia zinc finger in the development of innate-like CD8 + T cells. J Immunol. 2011;186(8):4573–8. 10.4049/jimmunol.1100037 . PubMed PMID: 21383242; PubMed Central PMCID: PMC3085897. PMC3085897 21383242

[83] Nayar R, Enos M, Prince A, Shin H, Hemmers S, Jiang J, kang, et al. TCR signaling via Tec kinase ITK and interferon regulatory factor 4 (IRF4) regulates CD8 + T-cell differentiation. Proc Natl Acad Sci U S A. 2012;109(41):E2794–2802. 10.1073/pnas.1205742109 . PubMed PMID: 23011795; PubMed Central PMCID: PMC3478592. PMC3478592 23011795

[84] Istaces N, Splittgerber M, Lima Silva V, Nguyen M, Thomas S, Le A, et al. EOMES interacts with RUNX3 and BRG1 to promote innate memory cell formation through epigenetic reprogramming. Nat Commun. 2019;10(1):3306. 10.1038/s41467-019-11233-6 . PubMed PMID: 31341159; PubMed Central PMCID: PMC6656725. PMC6656725 31341159

[85] Pokhrel S, Dileepan G, Leonard MR, Warren RT, Salem M, Yacapraro AJ, et al. Aiolos restricts the generation of antigen-inexperienced, virtual memory CD8 + T cells in mice. Nat Commun. 2025;17(1):839. 10.1038/s41467-025-67540-8 . PubMed PMID: 41392082; PubMed Central PMCID: PMC12827986. PMC12827986 41392082

[86] Paprckova D, Niederlova V, Moudra A, Drobek A, Pribikova M, Janusova S, et al. Self-reactivity of CD8 T-cell clones determines their differentiation status rather than their responsiveness in infections. Front Immunol. 2022;13:1009198. 10.3389/fimmu.2022.1009198 . PubMed PMID: 36275704; PubMed Central PMCID: PMC9582129. PMC9582129 36275704

[87] Schüler T, Hämmerling GJ, Arnold B. Cutting edge: IL-7-dependent homeostatic proliferation of CD8 + T cells in neonatal mice allows the generation of long-lived natural memory T cells. J Immunol. 2004;172(1):15–9. 10.4049/jimmunol.172. .1.15 PubMed PMID: 14688303. 14688303

[88] Le Campion A, Bourgeois C, Lambolez F, Martin B, Léaument S, Dautigny N, et al. Naive T cells proliferate strongly in neonatal mice in response to self-peptide/self-MHC complexes. Proc Natl Acad Sci U S A. 2002;99(7):4538–43. 10.1073/pnas.062621699 . PubMed PMID: 11917110; PubMed Central PMCID: PMC123683. PMC123683 11917110

[89] Surh CD, Sprent J. Regulation of mature T cell homeostasis. Semin Immunol. 2005;17(3):183–91. 10.1016/j.smim.2005. 02.007 PubMed PMID: 15826823. 15826823

[90] Marleau AM, Sarvetnick N. T cell homeostasis in tolerance and immunity. J Leukoc Biol. 2005;78(3):575–84. 10.1189/jlb.0105050 . PubMed PMID: 15894586. 15894586

[91] Jameson SC. T cell homeostasis: keeping useful T cells alive and live T cells useful. Semin Immunol. 2005;17(3):231–7. 10.1016/j.smim.2005.02. .003 PubMed PMID: 15826828. 15826828

[92] Goldrath AW, Bogatzki LY, Bevan MJ. Naive T cells transiently acquire a memory-like phenotype during homeostasis-driven proliferation. J Exp Med. 2000;192(4):557–64. 10.1084/jem.192.4.557 . PubMed PMID: 10952725; PubMed Central PMCID: PMC2193243. PMC2193243 10952725

[93] Hamilton SE, Wolkers MC, Schoenberger SP, Jameson SC. The generation of protective memory-like CD8 + T cells during homeostatic proliferation requires CD4 + T cells. Nat Immunol. 2006;7(5):475–81. 10.1038/ni1326 . PubMed PMID: 16604076. 16604076

[94] Renkema KR, Lee JY, Lee YJ, Hamilton SE, Hogquist KA, Jameson SC. IL-4 sensitivity shapes the peripheral CD8 + T cell pool and response to infection. J Exp Med. 2016;213(7):1319–29. 10.1084/jem.20151359 . PubMed PMID: 27298446; PubMed Central PMCID: PMC4925014. PMC4925014 27298446

[95] Baez NS, Cerbán F, Savid-Frontera C, Hodge DL, Tosello J, Acosta-Rodriguez E, et al. Thymic expression of IL-4 and IL-15 after systemic inflammatory or infectious Th1 disease processes induce the acquisition of innate characteristics during CD8 + T cell development. PLoS Pathog. 2019;15(1):e1007456. 10.1371/journal.ppat.1007456 . PubMed PMID: 30608984; PubMed Central PMCID: PMC6319713. PMC6319713 30608984

[96] Rolot M, Dougall AM, Chetty A, Javaux J, Chen T, Xiao X, et al. Helminth-induced IL-4 expands bystander memory CD8 + T cells for early control of viral infection. Nat Commun. 2018;9(1):4516. 10.1038/s41467-018-06978-5 . PubMed PMID: 30375396; PubMed Central PMCID: PMC6207712. PMC6207712 30375396

[97] Berg RE, Cordes CJ, Forman J. Contribution of CD8 + T cells to innate immunity: IFN-gamma secretion induced by IL-12 and IL-18. Eur J Immunol. 2002;32(10):2807–16. 10.1002/1521-4141(2002010)32:10<2807::AID-IMMU2807>3. 0.CO;2-0 PubMed PMID: 12355433. 12355433

[98] Lee H, Park SH, Shin EC. IL-15 in T-Cell Responses and Immunopathogenesis. Immune Netw. 2024;24(1):e11. 10.4110/in. .2024.24.e11 PubMed PMID: 38455459; PubMed Central PMCID: PMC10917573. 10.4110/in.2024.24.e11 PMC10917573 38455459

[99] Nakahira M, Ahn HJ, Park WR, Gao P, Tomura M, Park CS, et al. Synergy of IL-12 and IL-18 for IFN-gamma gene expression: IL-12-induced STAT4 contributes to IFN-gamma promoter activation by up-regulating the binding activity of IL-18-induced activator protein 1. J Immunol. 2002;168(3):1146–53. 10.4049/jimmunol.168.3.1146 . PubMed PMID: 11801649. 11801649

[100] Smith NL, Patel RK, Reynaldi A, Grenier JK, Wang J, Watson NB, et al. Developmental Origin Governs CD8 + T Cell Fate Decisions during Infection. Cell. 2018;174(1):117–e13014. 10.1016/j.cell.2018.05.029 . PubMed PMID: 29909981. 29909981

[101] Rudd BD, Venturi V, Li G, Samadder P, Ertelt JM, Way SS, et al. Nonrandom attrition of the naive CD8 + T-cell pool with aging governed by T-cell receptor:pMHC interactions. Proc Natl Acad Sci U S A. 2011;108(33):13694–9. 10.1073/pnas.1107594108 . PubMed PMID: 21813761; PubMed Central PMCID: PMC3158207. PMC3158207 21813761

[102] Marttila S, Jylhävä J, Pesu M, Hämäläinen S, Jylhä M, Hervonen A, et al. IL-7 concentration is increased in nonagenarians but is not associated with markers of T cell immunosenescence. Exp Gerontol. 2011;46(12):1000–2. .004 PubMed PMID: 21964236. 10.1016/j.exger.2011.09.004 21964236

[103] Decman V, Laidlaw BJ, Doering TA, Leng J, Ertl HCJ, Goldstein DR, et al. Defective CD8 T cell responses in aged mice are due to quantitative and qualitative changes in virus-specific precursors. J Immunol. 2012;188(4):1933–41. 10.4049/jimmunol.1101098 . PubMed PMID: 22246631; PubMed Central PMCID: PMC3320034. PMC3320034 22246631

[104] Moudra A, Niederlova V, Novotny J, Schmiedova L, Kubovciak J, Matejkova T, et al. Phenotypic and Clonal Stability of Antigen-Inexperienced Memory-like T Cells across the Genetic Background, Hygienic Status, and Aging. J Immunol. 2021;206(9):2109–21. 10.4049/jimmunol.2001028 . PubMed PMID: 33858960; PubMed Central PMCID: PMC7610663. PMC7610663 33858960

[105] Renkema KR, Li G, Wu A, Smithey MJ, Nikolich-Žugich J. Two separate defects affecting true naive or virtual memory T cell precursors combine to reduce naive T cell responses with aging. J Immunol. 2014;192(1):151–9. 10.4049/jimmunol.1301453 . PubMed PMID: 24293630; PubMed Central PMCID: PMC3925780. PMC3925780 24293630

[106] Al-Attar A, Presnell SR, Clasey JL, Long DE, Walton RG, Sexton M, et al. Human Body Composition and Immunity: Visceral Adipose Tissue Produces IL-15 and Muscle Strength Inversely Correlates with NK Cell Function in Elderly Humans. Front Immunol. 2018;9:440. 10.3389/fimmu.2018.00440 . PubMed PMID: 29559978; PubMed Central PMCID: PMC5845694. PMC5845694 29559978

[107] Quinn KM, Fox A, Harland KL, Russ BE, Li J, Nguyen THO, et al. Age-Related Decline in Primary CD8 + T Cell Responses Is Associated with the Development of Senescence in Virtual Memory CD8 + T Cells. Cell Rep. 2018;23(12):3512–24. 10.1016/j.celrep.2018.05. .057 PubMed PMID: 29924995. 29924995

[108] Borsa M, Barandun N, Gräbnitz F, Barnstorf I, Baumann NS, Pallmer K, et al. Asymmetric cell division shapes naive and virtual memory T-cell immunity during ageing. Nat Commun. 2021;12(1):2715. 10.1038/s41467-021-22954-y . PubMed PMID: 33976157; PubMed Central PMCID: PMC8113513. PMC8113513 33976157

[109] Quinn KM, Hussain T, Kraus F, Formosa LE, Lam WK, Dagley MJ, et al. Metabolic characteristics of CD8 + T cell subsets in young and aged individuals are not predictive of functionality. Nat Commun. 2020;11(1):2857. 10.1038/s41467-020-16633-7 . PubMed PMID: 32504069; PubMed Central PMCID: PMC7275080. PMC7275080 32504069

[110] Hussain T, Nguyen A, Daunt C, Thiele D, Pang ES, Li J, et al. Helminth Infection-Induced Increase in Virtual Memory CD8 T Cells Is Transient, Driven by IL-15, and Absent in Aged Mice. J Immunol. 2023;210(3):297–309. 10.4049/jimmunol.2200316 . PubMed PMID: 36524995. 36524995

[111] Hussain T, Nguyen A, Thiele D, Kannangara D, Huang Z, Pang ES, et al. LCMV-mediated loss of virtual memory CD8 T cells yields a functionally enhanced T cell subset. iScience. 2025;28(11):113893. 10.1016/j.isci.2025.113893 . PubMed PMID: 41280703; PubMed Central PMCID: PMC12639556. PMC12639556 41280703

[112] Morris SR, Chen B, Mudd JC, Panigrahi S, Shive CL, Sieg SF et al. Inflammescent CX3CR1 + CD57+CD8 + T cells are generated and expanded by IL-15. JCI Insight. 2020;5(11):e132963, 132963. 10.1172/jci.insight.132963 PubMed PMID: 32369455; PubMed Central PMCID: PMC7346586. PMC7346586 32369455

[113] Bez P, Santangeli E, Goldacker S, Salzer U, Warnatz K. Expansion of CD57 + CD8 T cells in common variable immunodeficiency with hepatopathy and CMV infection. Front Immunol. 2025;16:1577934. 10.3389/fimmu.2025.1577934 . PubMed PMID: 40496855; PubMed Central PMCID: PMC12149213. PMC12149213 40496855

[114] Freeman ML, Chakraborty A, O’Hare K, Haria A, Clagett B. Cytomegalovirus infection promotes the generation of IL-15-responsive CD57 + virtual memory CD8 T cells in people with and without HIV 2972. J Immunol. 2025;214(Supplement1):vkaf283825. 10.1093/jimmun/vkaf283.825.

[115] Khan N, Shariff N, Cobbold M, Bruton R, Ainsworth JA, Sinclair AJ, et al. Cytomegalovirus seropositivity drives the CD8 T cell repertoire toward greater clonality in healthy elderly individuals. J Immunol. 2002;169(4):1984–92. 10.4049/jimmunol.169.4. .1984 PubMed PMID: 12165524. 12165524

[116] Martin-Ruiz C, Hoffmann J, Shmeleva E, von Zglinicki T, Richardson G, Draganova L, et al. CMV-independent increase in CD27-CD28 + CD8+ EMRA T cells is inversely related to mortality in octogenarians. NPJ Aging Mech Dis. 2020;6:3. 10.1038/s41514-019-0041-y . PubMed PMID: 31993214; PubMed Central PMCID: PMC6972903. PMC6972903 31993214

[117] Alves NL, Arosa FA, van Lier RAW. IL-21 sustains CD28 expression on IL-15-activated human naive CD8 + T cells. J Immunol. 2005;175(2):755–62. 10.4049/jimmunol.175. .2.755 PubMed PMID: 16002671. 16002671

[118] Kuijpers TW, Vossen MT, Gent MR, Davin JC, Roos MT, Wertheim-van Dillen PM, et al. Frequencies of circulating cytolytic, CD45RA+CD27-, CD8 + T lymphocytes depend on infection with CMV. J Immunol. 2003;170(8):4342–8. 10.4049/jimmunol.170. .8.4342 PubMed PMID: 12682271. 12682271

[119] Almanzar G, Schwaiger S, Jenewein B, Keller M, Herndler-Brandstetter D, Würzner R, et al. Long-term cytomegalovirus infection leads to significant changes in the composition of the CD8 + T-cell repertoire, which may be the basis for an imbalance in the cytokine production profile in elderly persons. J Virol. 2005;79(6):3675–83. 2005 PubMed PMID: 15731261; PubMed Central PMCID: PMC1075718. 10.1128/JVI.79.6.3675-3683.2005 PMC1075718 15731261

[120] Griffiths SJ, Riddell NE, Masters J, Libri V, Henson SM, Wertheimer A, et al. Age-associated increase of low-avidity cytomegalovirus-specific CD8 + T cells that re-express CD45RA. J Immunol. 2013;190(11):5363–72. 10.4049/jimmunol.1203267 . PubMed PMID: 23636061; PubMed Central PMCID: PMC3736312. PMC3736312 23636061

[121] Brenchley JM, Karandikar NJ, Betts MR, Ambrozak DR, Hill BJ, Crotty LE, et al. Expression of CD57 defines replicative senescence and antigen-induced apoptotic death of CD8 + T cells. Blood. 2003;101(7):2711–20. 10.1182/blood. -2002-07-2103 PubMed PMID: 12433688. 12433688

[122] Rufer N, Zippelius A, Batard P, Pittet MJ, Kurth I, Corthesy P, et al. Ex vivo characterization of human CD8 + T subsets with distinct replicative history and partial effector functions. Blood. 2003;102(5):1779–87. 10.1182/blood. -2003-02-0420 PubMed PMID: 12750165. 12750165

[123] Schaum N, Lehallier B, Hahn O, Pálovics R, Hosseinzadeh S, Lee SE, et al. Ageing hallmarks exhibit organ-specific temporal signatures. Nature. 2020;583(7817):596–602. 10.1038/s41586-020-2499. y PubMed PMID: 32669715; PubMed Central PMCID: PMC7757734. PMC7757734 32669715

[124] Redruello-Romero A, Benitez-Cantos MS, Lopez-Perez D, García-Rubio J, Tamayo F, Pérez-Bartivas D, et al. Human adipose tissue as a major reservoir of cytomegalovirus-reactive T cells. Front Immunol. 2023;14:1303724. 10.3389/fimmu.2023.1303724 . PubMed PMID: 38053998; PubMed Central PMCID: PMC10694288. PMC10694288 38053998

[125] Couturier J, Suliburk JW, Brown JM, Luke DJ, Agarwal N, Yu X, et al. Human adipose tissue as a reservoir for memory CD4 + T cells and HIV. AIDS. 2015;29(6):667–74. 10.1097/QAD.0000000000000599 . PubMed PMID: 25849830; PubMed Central PMCID: PMC4389783. PMC4389783 25849830

[126] Han SJ, Glatman Zaretsky A, Andrade-Oliveira V, Collins N, Dzutsev A, Shaik J, et al. White Adipose Tissue Is a Reservoir for Memory T Cells and Promotes Protective Memory Responses to Infection. Immunity. 2017;47(6):1154–e11686. PubMed PMID: 29221731; PubMed Central PMCID: PMC5773068. 10.1016/j.immuni.2017.11.009 PMC5773068 29221731

[127] Bruno MEC, Mukherjee S, Powell WL, Mori SF, Wallace FK, Balasuriya BK, et al. Accumulation of γδ T cells in visceral fat with aging promotes chronic inflammation. Geroscience. 2022;44(3):1761–78. 10.1007/s11357-022-00572-w . PubMed PMID: 35477832; PubMed Central PMCID: PMC9213615. PMC9213615 35477832

[128] Lumeng CN, Liu J, Geletka L, Delaney C, Delproposto J, Desai A, et al. Aging is associated with an increase in T cells and inflammatory macrophages in visceral adipose tissue. J Immunol. 2011;187(12):6208–16. 10.4049/jimmunol.1102188 . PubMed PMID: 22075699; PubMed Central PMCID: PMC3237772. PMC3237772 22075699

[129] Weisberg SP, McCann D, Desai M, Rosenbaum M, Leibel RL, Ferrante AW. Obesity is associated with macrophage accumulation in adipose tissue. J Clin Invest. 2003;112(12):1796–808. doi:10.1172/JCI19246 PubMed PMID: 14679176; PubMed Central PMCID: PMC296995. 10.1172/JCI19246 PMC296995 14679176

[130] Nishimura S, Manabe I, Nagasaki M, Eto K, Yamashita H, Ohsugi M, et al. CD8 + effector T cells contribute to macrophage recruitment and adipose tissue inflammation in obesity. Nat Med. 2009;15(8):914–20. 10. 1038/nm.1964 PubMed PMID: 19633658. 10.1038/nm.1964 19633658

[131] Terrabuio E, Zenaro E, Constantin G. The role of the CD8 + T cell compartment in ageing and neurodegenerative disorders. Front Immunol. 2023;14:1233870. 10.3389/fimmu. 2023.1233870 PubMed PMID: 37575227; PubMed Central PMCID: PMC10416633. PMC10416633 37575227

[132] Shan L, van den Hoogen LL, Meeldijk J, Kok HM, Jongeneel LH, Boes M, et al. Increased intra-articular granzyme M may trigger local IFN-λ1/IL-29 response in rheumatoid arthritis. Clin Exp Rheumatol. 2020;38(2):220–6. 10.55563/clinexprheumatol/ffb. 107 PubMed PMID: 31172927. 31172927

[133] Zheng Y, Zhao J, Shan Y, Guo S, Schrodi SJ, He D. Role of the granzyme family in rheumatoid arthritis: Current Insights and future perspectives. Front Immunol. 2023;14:1137918. 10.3389/fimmu.2023.1137918 . PubMed PMID: 36875082; PubMed Central PMCID: PMC9977805. PMC9977805 36875082

[134] Ono M, Bolland S, Tempst P, Ravetch JV. Role of the inositol phosphatase SHIP in negative regulation of the immune system by the receptor Fc(gamma)RIIB. Nature. 1996;383(6597):263–6. 10.1038/383263a0 . PubMed PMID: 8805703. 8805703

[135] Nitschke L, Carsetti R, Ocker B, Köhler G, Lamers MC. CD22 is a negative regulator of B-cell receptor signalling. Curr Biol. 1997;7(2):133–43. 10.1016/s0960-9822(. 06)00057-1 PubMed PMID: 9016707. 9016707

[136] Starbeck-Miller GR, Badovinac VP, Barber DL, Harty JT. Cutting edge: Expression of FcγRIIB tempers memory CD8 T cell function in vivo. J Immunol. 2014;192(1):35–9. 10.4049/jimmunol.1302232 . PubMed PMID: 24285839; PubMed Central PMCID: PMC3874719. PMC3874719 24285839

[137] Bennion KB, Tariq M, Wyatt MM, Duneton C, Baecher KM, Paulos CM, et al. FcγRIIB expressed on CD8 + T cells limits responsiveness to PD-1 checkpoint inhibition in cancer. Sci Transl Med. 2023;15(710):eadd1868. 10.1126/scitranslmed.add1868 . PubMed PMID: 37611081; PubMed Central PMCID: PMC11325091. PMC11325091 37611081

[138] Morris AB, Adelman MW, Bennion KB, Martinez CD, McCook KM, Woodworth MH, et al. Fgl2 regulates FcγRIIB+CD8 + T cell responses during infection. JCI Insight. 2025;10(7):e186259. 10.1172/jci.insight.186259 . PubMed PMID: 40197366; PubMed Central PMCID: PMC11981615. PMC11981615 40197366

[139] Baecher KM, Ford ML. Intersection of FcγRIIB, the microbiome, and checkpoint inhibitors in antitumor immunity. Cancer Immunol Immunother. 2021;70(12):3397–404. 10.1007/s00262-021-03004-4 . PubMed PMID: 34241677; PubMed Central PMCID: PMC10992943. PMC10992943 34241677

[140] Morris AB, Farley CR, Pinelli DF, Adams LE, Cragg MS, Boss JM, et al. Signaling through the Inhibitory Fc Receptor FcγRIIB Induces CD8 + T Cell Apoptosis to Limit T Cell Immunity. Immunity. 2020;52(1):136–e1506. 10.1016/j.immuni.2019.12.006 . PubMed PMID: 31940267; PubMed Central PMCID: PMC7326381. PMC7326381 31940267

[141] Srinivasan S, Mishra S, Fan KKH, Wang L, Im J, Segura C, et al. Age-Dependent Bi-Phasic Dynamics of Ly49 + CD8+ Regulatory T Cell Population. Aging Cell. 2025;24(4):e14461. 10.1111/acel.14461 . PubMed PMID: 39696807; PubMed Central PMCID: PMC11984669. PMC11984669 39696807

[142] Yu L, Wan Q, Liu Q, Fan Y, Zhou Q, Skowronski AA, et al. IgG is an aging factor that drives adipose tissue fibrosis and metabolic decline. Cell Metab. 2024;36(4):793–e8075. 10.1016/j.cmet.2024.01.015 . PubMed PMID: 38378001; PubMed Central PMCID: PMC11070064. PMC11070064 38378001

[143] Walters H. IgG is an early driver of aging. Nat Aging. 2024;4(3):279. 10.1038/s43587-024-00602-6 . PubMed PMID: 38472455. 38472455

[144] Thiele D, Nguyen A, Hussain T, Barugahare A, Bennett T, Li J, et al. Exploring the Role of TVM Cells in Age-Related CD8 + T Cell Dysfunction through ATAC-seq and RNA-seq Analysis. J Immunol. 2023;210(Supplement1):8302–8302. 10.4049/jimmunol.210.Supp.83.02.

[145] Kasakovski D, Zeng X, Lai J, Yu Z, Yao D, Chen S, et al. Characterization of KIR + NKG2A + Eomes- NK-like CD8 + T cells and their decline with age in healthy individuals. Cytometry B Clin Cytom. 2021;100(4):467–75. 10.1002/cyto.b.21945 . PubMed PMID: 32830898. 32830898

[146] Jacomet F, Cayssials E, Basbous S, Levescot A, Piccirilli N, Desmier D, et al. Evidence for eomesodermin-expressing innate-like CD8(+) KIR/NKG2A(+) T cells in human adults and cord blood samples. Eur J Immunol. 2015;45(7):1926–33. 10.1002/eji.201545539 . PubMed PMID: 25903796. 25903796

[147] Wills MR, Carmichael AJ, Weekes MP, Mynard K, Okecha G, Hicks R, et al. Human virus-specific CD8 + CTL clones revert from CD45ROhigh to CD45RAhigh in vivo: CD45RAhighCD8 + T cells comprise both naive and memory cells. J Immunol. 1999;162(12):7080–7. PubMed PMID: 10358151. 10358151

[148] Van Kaer L. Innate and virtual memory T cells in man. Eur J Immunol. 2015;45(7):1916–20. 10.1002/eji.201545761 . PubMed PMID: 26013879; PubMed Central PMCID: PMC4540369. PMC4540369 26013879

[149] Geginat J, Lanzavecchia A, Sallusto F. Proliferation and differentiation potential of human CD8 + memory T-cell subsets in response to antigen or homeostatic cytokines. Blood. 2003;101(11):4260–6. 10.1182/blood-2002. -11-3577 PubMed PMID: 12576317. 12576317

[150] Herndler-Brandstetter D, Schwaiger S, Veel E, Fehrer C, Cioca DP, Almanzar G, et al. CD25-expressing CD8 + T cells are potent memory cells in old age. J Immunol. 2005;175(3):1566–74. 10.4049/jimmunol.175.3.1566 . PubMed PMID: 16034095. 16034095

[151] Schwaiger S, Wolf AM, Robatscher P, Jenewein B, Grubeck-Loebenstein B. IL-4-producing CD8 + T cells with a CD62L++(bright) phenotype accumulate in a subgroup of older adults and are associated with the maintenance of intact humoral immunity in old age. J Immunol. 2003;170(1):613–9. 10.4049/jimmunol.170.1. .613 PubMed PMID: 12496450. 12496450

[152] Herndler-Brandstetter D, Veel E, Laschober GT, Pfister G, Brunner S, Walcher S, et al. Non-regulatory CD8 + CD45RO+CD25 + T-lymphocytes may compensate for the loss of antigen-inexperienced CD8 + CD45RA + T-cells in old age. Biol Chem. 2008;389(5):561–8. 10.1515/bc.2008.052 . PubMed PMID: 18953723. 18953723

